# Supplementary material for: Sensitivity of temperature-based time since death estimation on measurement location
Source: Int J Legal Med. 2023 Jun 19;137(6):1815–37. doi: 10.1007/s00414-023-03040-y (PMC10567880; doi:10.1007/s00414-023-03040-y)
Supplement: Supplementary file 1 — Supplementary file1 (DOCX 3705 KB) [file 414_2023_3040_MOESM1_ESM.docx]

ELECTRONIC SUPPLEMENTARY

**Sensitivity of temperature-based time since death estimation on measurement location**

J. Shanmugam Subramaniam^1^, M. Hubig^1^, H. Muggenthaler^1^, S. Schenkl^1^, J. Ullrich^2^, G. Pourtier^2^, M. Weiser^2^, G. Mall^1^

^1^ Institute of Forensic Medicine, Jena University Hospital - Friedrich Schiller University Jena, Am Klinikum 1, 07747 Jena, Germany

^2^ Zuse Institute Berlin, Takustraße 7, 14195 Berlin, Germany

📧 Michael Hubig, Michael.Hubig@med.uni-jena.de

**Quantification method for TDE deviations**

TOD deviation from the central position C is quantified by L2 and maximum norm and the mathematical application of these two norms in TOD estimation is discussed in this section. Let the time interval [a, b] be given for body cooling and let a = 1 hour be the starting point of cooling and b = 45 hours be the end point of cooling. Let further T_1_(t) and T_2_(t) be two cooling curves defined on the interval [a, b]. The ambient temperature T_A_ is assumed constant in space and time outside the body. The ***reference curve*** T_R_(t) for T_1_(t) and T_2_(t) is constructed as the pointwise mean of T_1_(t) and T_2_(t):

(6) $\forall t\in\left[ a,b \right]: T_{R}\left( t \right):=(T_{1}\left( t \right)+T_{2}(t))/2$

Let T_0_ := T_R_(a) be the initial temperature of the reference curve T_R_(t). Now the reference curve T_R_(t) can be normalized to the function Q(t) taking values in the real number’s interval [0, 1]:

(7) $\forall t\in\left[ a,b \right]: Q\left( t \right):=(T_{R}\left( t \right)-T_{A})/(T_{0}-T_{A})$

This makes it possible to define the Q-intervals Q_1_ to Q_4_ and their left and right boundaries q_0_, … , q_4_ by:

(8) $q_{0}:=1, q_{1}:=0.5, q_{2}:=0.3, q_{3}:=0.2, q_{4}:=0.1$

(9) $\forall1\leq n\leq4: Q_{n}:=]q_{n-1},q_{n}]$

Assuming T_R_(t) to be strictly monotonically decreasing, we can uniquely map the Q-interval boundaries q_n_ to the respective temperature values T_R(4-n)_.

(10) $\forall0\leq n\leq4: T_{R(4-n)}:=q_{n}\cdot\left( T_{0}-T_{A} \right)+T_{A}$

Since the two cooling curves T_1_ and T_2_ are monotonically decreasing it is possible to define their inverse curves t_1_, t_2_, called the **(absolute) time since death (TSD) estimation result curves** w.r.t. the cooling curves T_1_, T_2_ on the domain [T_MIN_,T_MAX_] which is the range of the reference curve T_R_ on the temperature axis by:

(11) $t_{1}:[T_{MIN},T_{MAX}]\to IR:(T\to t_{1}\left( T \right):=T_{1}^{-1}\left( T \right))$

(12) $t_{2}:[T_{MIN},T_{MAX}]\to IR:(T\to t_{2}\left( T \right):=T_{2}^{-1}\left( T \right))$

Moreover we define the **relative TSD** t_1_*, t_2_* w.r.t. the cooling curves T_1_,T_2_:

(13) ${t_{1}}^{*}:[T_{MIN},T_{MAX}]\to IR:(T\to{t_{1}}^{*}\left( T \right):=\frac{t_{1}\left( T \right)}{t_{1}\left( T \right)+t_{2}\left( T \right)})$

(14) ${t_{2}}^{*}:[T_{MIN},T_{MAX}]\to IR:(T\to{t_{2}}^{*}\left( T \right):=\frac{t_{1}\left( T \right)}{t_{1}\left( T \right)+t_{2}\left( T \right)})$

The absolute and the relative time functions t_1_, t_2_ and t_1_*, t_2_* are frequently concatenated with the reference curve T_R_, thus giving t_1_(T_R_(t)), t_2_(T_R_(t)) and t_1_*(T_R_(t)), t_2_*(T_R_(t)) in the following definitions. For short those concatenations will be abbreviated t_1_(t), t_2_(t) and t_1_*(t), t_2_*(t). If the point t in time is clear, we will even write t_1_, t_2,_ t_1_*, t_2_*. We will now quantify the distance between the cooling curves T_1_ and T_2_, which are actually constructed as distances between the inverse curves t_1_ and t_2_. The reason for this somehow twisted construction lies in our prior interest in time differences because our research’s number one target is *time since death*. All of the temperature curve - distance measures to be defined in the following are constructed using the maximum norm MAX or the L_2_ norm, which are shown here for continuous functions first. The maximum domain of the reference curve T_R_ on the time axis is the interval [t_MIN_,t_MAX_] which is taken therefore as the domain for maximum-finding in our definition :

(15) $\left\| t_{1}\left( T_{R}\left( t \right) \right)- t_{2}(T_{R}(t)) \right\|_{MAX}:=\max_{t\epsilon\left[ t_{MIN}{,t}_{MAX} \right]} \left| t_{1}\left( T_{R}\left( t \right) \right)- t_{2}(T_{R}(t)) \right|$

(16) $\left\| {t_{1}}^{*}\left( T_{R}\left( t \right) \right)- {t_{2}}^{*}(T_{R}(t)) \right\|_{MAX}:=\max_{t\epsilon\left[ t_{MIN}{,t}_{MAX} \right]} \left| {t_{1}}^{*}\left( T_{R}\left( t \right) \right)- {t_{2}}^{*}(T_{R}(t)) \right|$

(17) $\left\| t_{1}\left( T_{R}\left( t \right) \right)- t_{2}(T_{R}(t)) \right\|_{L2}:=\sqrt{\frac{1}{t_{MAX}-t_{MIN}}\cdot\int_{t_{MIN}}^{t_{MAX}} \left( t_{1}\left( T_{R}\left( t \right) \right)- t_{2}(T_{R}(t)) \right)^{2}dt}$

(18) $\left\| {t_{1}}^{*}\left( T_{R}\left( t \right) \right)- {t_{2}}^{*}(T_{R}(t)) \right\|_{L2}:=\sqrt{\frac{1}{t_{MAX}-t_{MIN}}\cdot\int_{t_{MIN}}^{t_{MAX}} \left( {t_{1}}^{*}\left( T_{R}\left( t \right) \right)- {t_{2}}^{*}(T_{R}(t)) \right)^{2}dt}$

We will give now equivalents of the four definitions (15) – (18) in terms of real measured temperature curves T_i_(t). Each curve T_i_(t) of is represented as a finite series of real measurement values (T_i_^1^, … , T_i_^N^) on a finite (regular) grid of time values (t^1^, … , t^N^) which is the same for both of the curves. The first point t^1^ of the time grid is identical to the starting time of cooling computation, while the last point t^N^ marks the end of the cooling computation interval. Let now be:

(19) $T_{MAX}:=min\left\{ T_{1}^{1},T_{2}^{1} \right\}$

(20) $T_{MIN}:=max\left\{ T_{1}^{N},T_{2}^{N} \right\}$

Now we have constructed the range of our reference curve T_R_(t). The limits of its domain of definition [t_MIN_,t_MAX_] can then be computed easily:

(21) $t_{MIN}:={T_{R}}^{-1}(T_{MAX})$

(22) $t_{MAX}:={T_{R}}^{-1}(T_{MIN})$

Now let K be a natural number and let (t^1^, … , t^K^) be a regular time grid on the interval [t_MIN_,t_MAX_] with t^1^ := t_MIN_ and t^K^ := t_MAX_. Let further be (T^1^, … , T^K^) the corresponding temperature grid with T^1^ ≤ … ≤ T^K^ and T^k^ := T_R_(t^k^) for all k = 1, … , K and T^K^ := T_MAX_. The the time - grid’s width Δt is:

(23) $\Delta t:=\frac{t_{MAX}-t_{MIN}}{K-1}$

Let further be t_qn_ the inverse image of T_Rn_ under the reference function T_R_ for all n = 0, … , 4:

(24) $\forall0\leq n\leq4: t_{qn}:=T_{R}^{-1}\left( T_{R(4-n)} \right)$

The five points t_qn_ on the time scale constitute the endpoints of four time intervals t_Q1_, … , t_Q4_:

(25) $\forall1\leq n\leq4: t_{Qn}:=]t_{qn-1},t_{qn}]$

We will now redefine our four measures (15) - (18) quantifying the distance between the cooling curves T_1_ and T_2_ in terms of the real samples (t^1^, … , t^K^) and (T_i_^1^, … , T_i_^K^). The four distance measures are the ***(absolute)*** ***maximum distance*** D_MAX_ (T_1_, T_2_), the ***relative*** ***maximum distance*** d_MAX_ (T_1_, T_2_), the ***(absolute)*** ***L2 distance*** D_L2_ (T_1_, T_2_), the ***relative*** ***L2 distance*** d_L2_ (T_1_, T_2_). Each of the four distances is defined in a global version on the whole time interval [t_MIN_, t_MAX_] and in four local ones residing on one of the intersection t_Qj_ ∩ [t_MIN_, t_MAX_] on the time axis each. For all 1 ≤ i ≤ 4 the number of t^k^ lying in t_Qj_ ∩ [t_MIN_, t_MAX_] is denoted by K_i_

The ***(absolute global)*** ***maximum distance*** D_MAX_(T_1_, T_2_) is defined as:

(26) $D_{MAX}\left( T_{1},T_{2} \right):={max}_{1\leq k\leq K}|t_{1}\left( T_{R}\left( t^{k} \right) \right)-t_{2}\left( T_{R}\left( t^{k} \right) \right)|$

while the ***(absolute)*** ***Q_i_-local maximum distance*** D_MAX,Qi_(T_1_, T_2_) is defined for i = 1, … , 4:

(27) $D_{MAX,Q_{i}}\left( T_{1},T_{2} \right):={max}_{1\leq k\leq K_{i}, t^{k}\in t_{Qi} \cap[t_{MIN}, t_{MAX}]}|t_{1}\left( T_{R}\left( t^{k} \right) \right)-t_{2}\left( T_{R}\left( t^{k} \right) \right)|$

The ***(absolute global) L_2_-distance*** D_L2_(T_1_, T_2_) is determined by the following formula which is something like the mean pointwise square distance:

(28) $D_{L2}\left( T_{1},T_{2} \right):=\sqrt{\frac{1}{K-1}\cdot\sum_{1\leq k\leq K} \left( t_{1}\left( T_{R}\left( t^{k} \right) \right)-t_{2}\left( T_{R}\left( t^{k} \right) \right) \right)^{2}}$

and the ***(absolute)*** ***Q_i_-local L_2_ distance*** D_L2,Qi_(T_1_, T_2_) is determined by:

(29) $D_{L2,Q_{i}}\left( T_{1},T_{2} \right):=\sqrt{\frac{1}{K_{i}-1}\cdot\sum_{t^{k}\in t_{Qi}\cap[t_{MIN}, t_{MAX}]} \left( t_{1}\left( T_{R}\left( t^{k} \right) \right)-t_{2}\left( T_{R}\left( t^{k} \right) \right) \right)^{2}}$

The ***relative (global) L_2_-distance*** d_L2_(T_1_, T_2_) is defined:

(30) $d_{L2}\left( T_{1},T_{2} \right):=\sqrt{\frac{1}{K-1}\cdot\sum_{1\leq k\leq K} \left( {t_{1}}^{*}\left( T_{R}\left( t^{k} \right) \right)-{t_{2}}^{*}\left( T_{R}\left( t^{k} \right) \right) \right)^{2}}$

whereas the ***(relative)*** ***Q_i_-local L_2_ distance*** d_L2,Qi_(T_1_, T_2_) is:

(31) $d_{L2,Q_{i}}\left( T_{1},T_{2} \right):=\sqrt{\frac{1}{K_{i}-1}\cdot\sum_{t^{k}\in t_{Qi}\cap[t_{MIN}, t_{MAX}]} \left( {t_{1}}^{*}\left( T_{R}\left( t^{k} \right) \right)-{t_{2}}^{*}\left( T_{R}\left( t^{k} \right) \right) \right)^{2}}$

It is well known and easy to see that D_L2_(T_1_, T_2_) as defined in (28) is in good approximation for large K identical to the usual continuous definition of the L_2_-distance as in (17) between continuous functions:

(32) $D_{L2}\left( T_{1},T_{2} \right)=\sqrt{\frac{1}{K-1}\cdot\sum_{1\leq k\leq K} \left( t_{1}\left( T_{R}\left( t^{k} \right) \right)-t_{2}\left( T_{R}\left( t^{k} \right) \right) \right)^{2}}$

$$=\sqrt{\frac{1}{\left( K-1 \right)\cdot\Delta t}\cdot\Delta t\cdot\sum_{1\leq k\leq K} \left( {t_{1}}^{*}\left( T_{R}\left( t^{k} \right) \right)-t_{2}\left( T_{R}\left( t^{k} \right) \right) \right)^{2}}$$

$$=\sqrt{\frac{1}{t_{MAX}-t_{MIN}}\cdot\sum_{1\leq k\leq K} {\Delta t\cdot\left( {t_{1}}^{*}\left( T_{R}\left( t^{k} \right) \right)-t_{2}\left( T_{R}\left( t^{k} \right) \right) \right)}^{2}}$$

$$\approx\sqrt{\frac{1}{t_{MAX}-t_{MIN}}\cdot\int_{t_{MIN}}^{t_{MAX}} \left( t_{1}\left( T_{R}\left( t \right) \right)-t_{2}\left( T_{R}\left( t \right) \right) \right)^{2}dt}$$

$$=\left\| t_{1}\left( T_{R}\left( t \right) \right)- t_{2}(T_{R}(t)) \right\|_{L2}$$

Moreover the following relation, which is shown here for (26) and (28), between L2 norms and maximum norms is well known:

(33) $\left\| t_{1}\left( T_{R}\left( t \right) \right)- t_{2}(T_{R}(t)) \right\|_{L2}$

$=\sqrt{\frac{1}{t_{MAX}-t_{MIN}}\cdot\int_{t_{MIN}}^{t_{MAX}} \left( t_{1}\left( T_{R}\left( t \right) \right)- t_{2}(T_{R}(t)) \right)^{2}dt}$

$$\leq\sqrt{\frac{1}{t_{MAX}-t_{MIN}}\cdot\int_{t_{MIN}}^{t_{MAX}} \left( \max_{s\in\left[ t_{MIN},t_{MAX} \right]} \left| t_{1}\left( T_{R}\left( s \right) \right)- t_{2}(T_{R}(s)) \right| \right)^{2}dt}$$

$$\leq\max_{s\in\left[ t_{MIN},t_{MAX} \right]} \left| t_{1}\left( T_{R}\left( s \right) \right)- t_{2}\left( T_{R}\left( s \right) \right) \right|\cdot\sqrt{\frac{1}{t_{MAX}-t_{MIN}}\cdot\int_{t_{MIN}}^{t_{MAX}} 1dt}$$

$$\leq\max_{s\in\left[ t_{MIN},t_{MAX} \right]} \left| t_{1}\left( T_{R}\left( s \right) \right)- t_{2}(T_{R}(s)) \right|$$

$\leq\left\| {t_{1}}^{*}\left( T_{R}\left( t \right) \right)- {t_{2}}^{*}(T_{R}(t)) \right\|_{MAX}$

**Results**

The TDE deviation from the actual measurement position is measured by L2 norm (D_L2_, d_L2_) and maximum norm (D_MAX_, d_MAX_) for CTM, CMS, and CM models depending on ambient temperature as well as measurement radius R. Tab. 2 gives the direction of the minimum and maximum TDE deviation for each considered model (CTM, CMS, and CM) evaluated depending on ambient temperature T_A_ and measurement radius R. For an instance, the maximum and minimum TDE deviation in the absolute maximum norm D_max_ in Y and X direction respectively in both CTM and CMS model. However, in CM model the maximum and minimum TDE deviation in the absolute maximum norm is seen in Z and X direction respectively whereas in d_L2_ and d_MAX_ the maximum and minimum TDE deviation is observed in Y and X direction respectively in CM.

| Model | TDE - deviation | D_L2_ | d_L2_ | D_MAX_ | d_MAX_ |
| --- | --- | --- | --- | --- | --- |
| CTM – TA = 5° C, 15° C, 25° C | Min | X | X | X | X |
|  | Max | Y | Y | Y | Y |
| CTM – R = 0.5 cm, 1 cm, 2 cm | Min | X | X | X | X |
|  | Max | Y | Y | Y | Y |
| CMS - TA = 5° C, 15° C, 25° C | Min | X | X | X | X |
|  | Max | Y | Y | Y | Y |
| CMS - R = 0.5 cm, 1 cm, 2 cm | Min | X | X | X | X |
|  | Max | Y | Y | Y | Y |
| CM - TA = 5° C, 15° C, 25° C | Min | X | X | X | X |
|  | Max | Z | Y | Z | Y |
| CM - R = 0.5 cm, 1 cm, 2 cm | Min | X | X | X | X |
|  | Max | Z | Y | Z | Y |

**Tab. 1:** Min- and max-deviation of measures D_L2_, d_L2_, D_MAX_, d_MAX_ for CM, CMS, CTM models for ambient temperatures T_A_ = 5° C, 15° C, 25° C and for measurement-location radii R = 0.5 cm, 1 cm, 2 cm.

TDE deviation measured by D_L2_, d_L2_, D_MAX_, d_MAX_ on CTM, CMS, CM model depending on ambient temperature T_A_ and measurement radius R is represented in Figure 5 to 28.


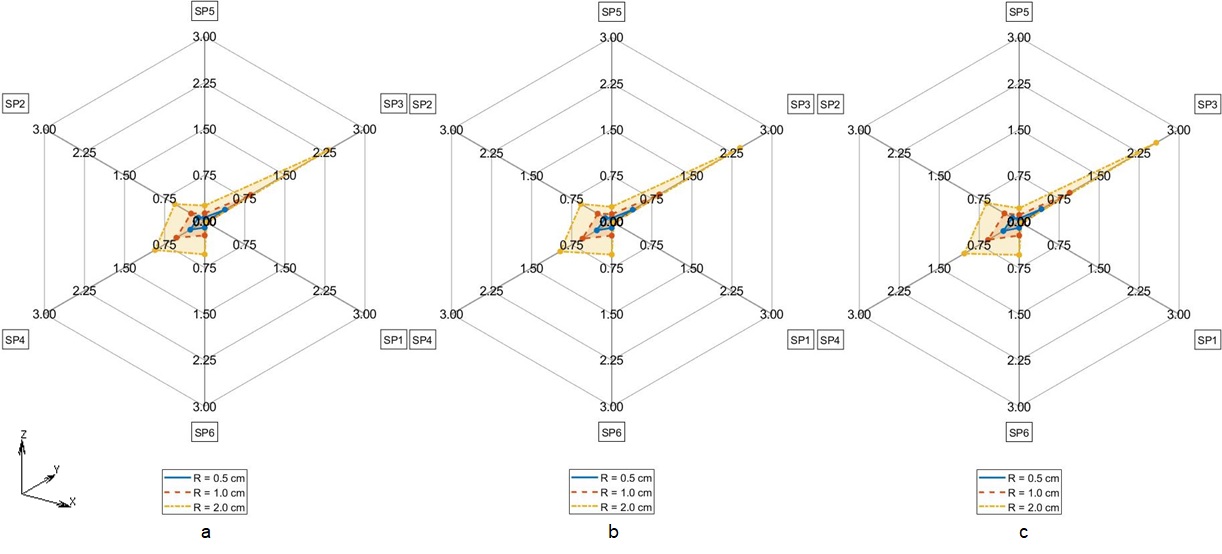
**Fig. 11:** TDE deviation in D_L2_ for CTM for R = 0.5 cm (Solid line), R = 1.0 cm (Dashed line), R = 2 cm (Dash-dotted line) a. TA = 5° C, b. TA = 15° C, c. TA = 25° C


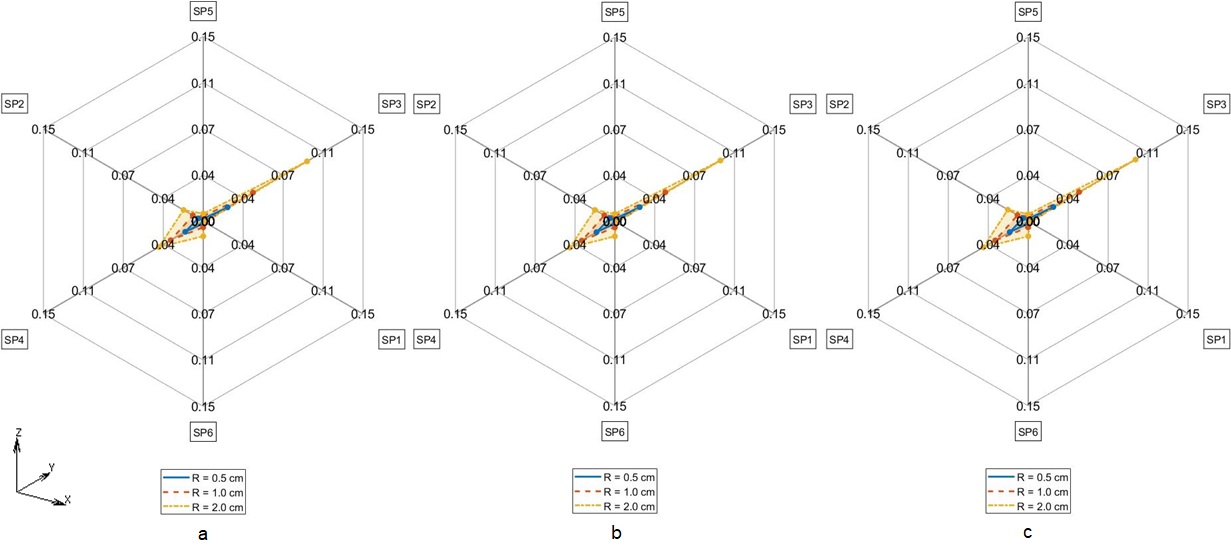
**Fig. 12:** TDE deviation in d_L2_ for CTM for R = 0.5 cm (Solid line), R = 1.0 cm (Dashed line), R = 2 cm (Dash-dotted line) a. TA = 5° C, b. TA = 15° C, c. TA = 25° C


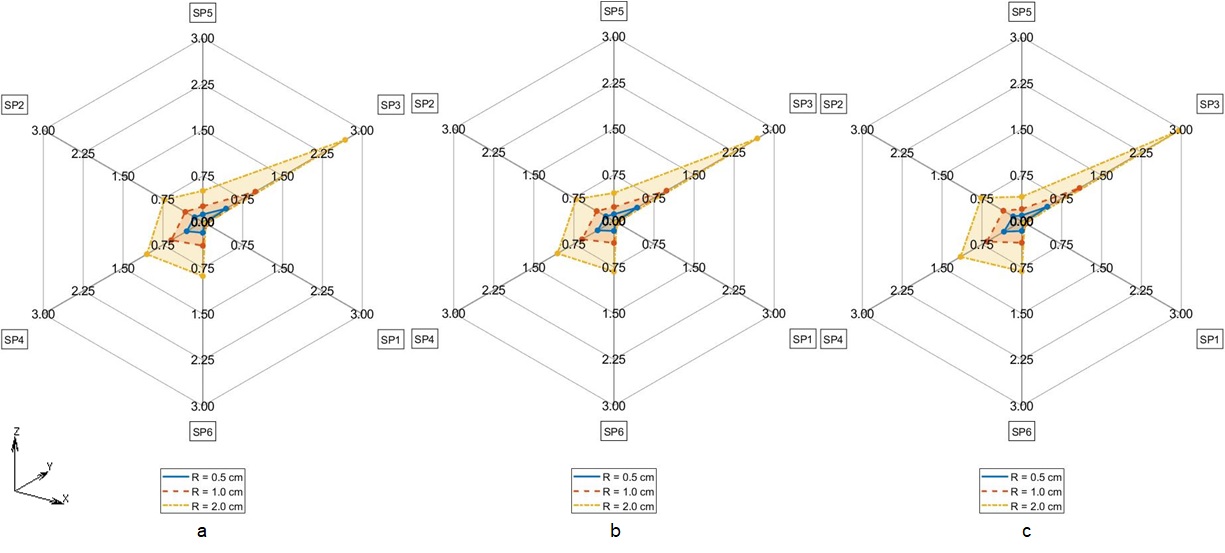


**Fig. 13:** TDE deviation in D_MAX_ for CTM for R = 0.5 cm (Solid line), R = 1.0 cm (Dashed line), R = 2 cm (Dash-dotted line) a. TA = 5° C, b. TA = 15° C, c. TA = 25° C


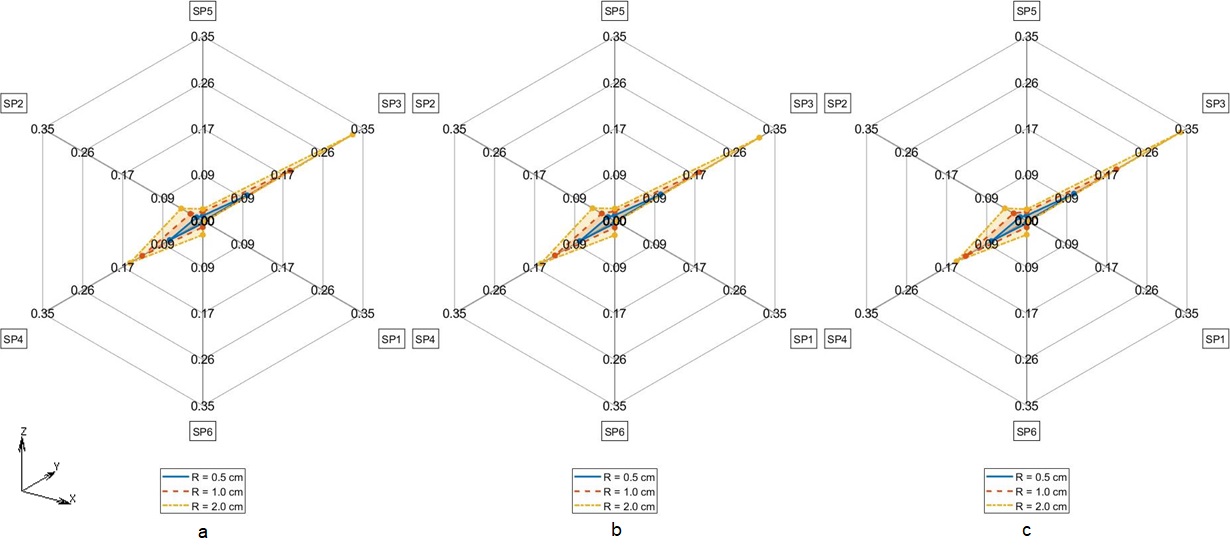


**Fig. 14:** TDE deviation in d_MAX_ for CTM for R = 0.5 cm (Solid line), R = 1.0 cm (Dashed line), R = 2 cm (Dash-dotted line) a. TA = 5° C, b. TA = 15° C, c. TA = 25° C


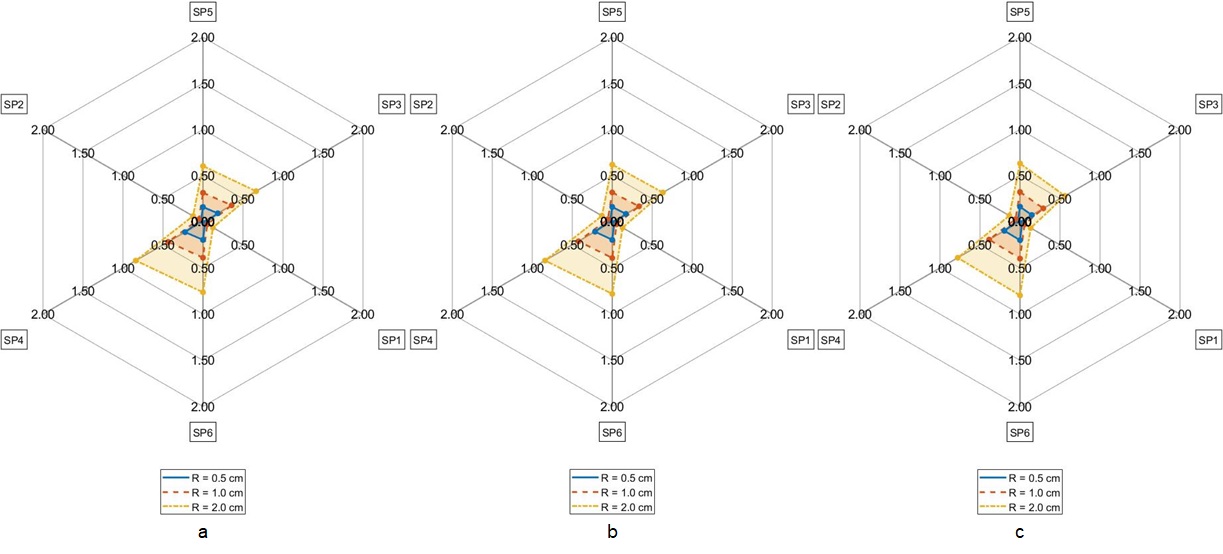


**Fig. 15:** TDE deviation in D_L2_ for CMS for R = 0.5 cm (Solid line), R = 1.0 cm (Dashed line), R = 2 cm (Dash-dotted line) a. TA = 5° C, b. TA = 15° C, c. TA = 25° C


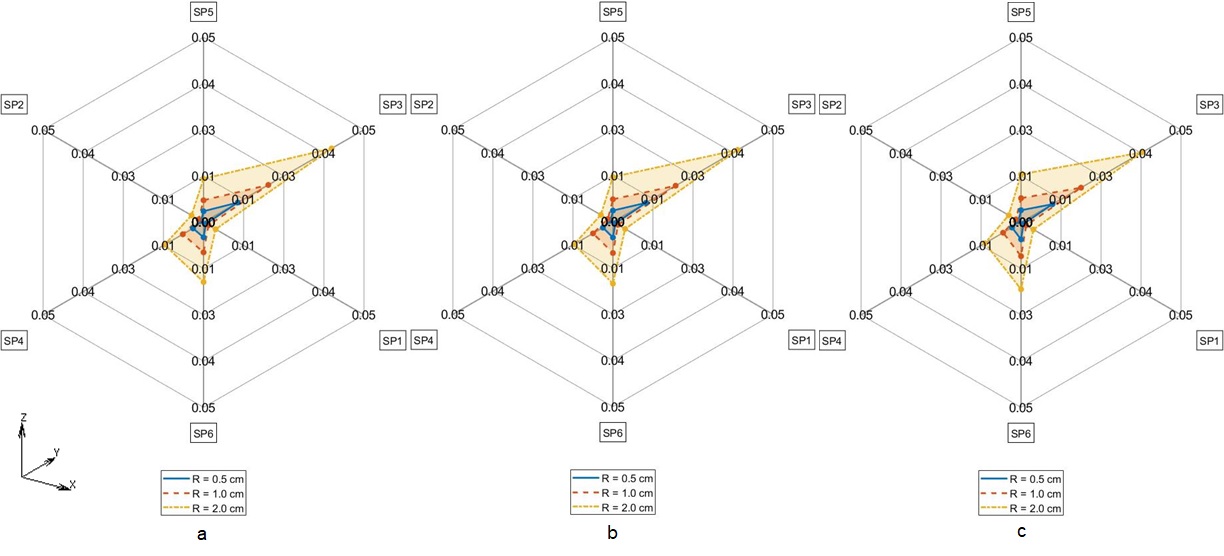


**Fig. 16:** TDE deviation in d_L2_ for CMS for R = 0.5 cm (Solid line), R = 1.0 cm (Dashed line), R = 2 cm (Dash-dotted line) a. TA = 5° C, b. TA = 15° C, c. TA = 25° C


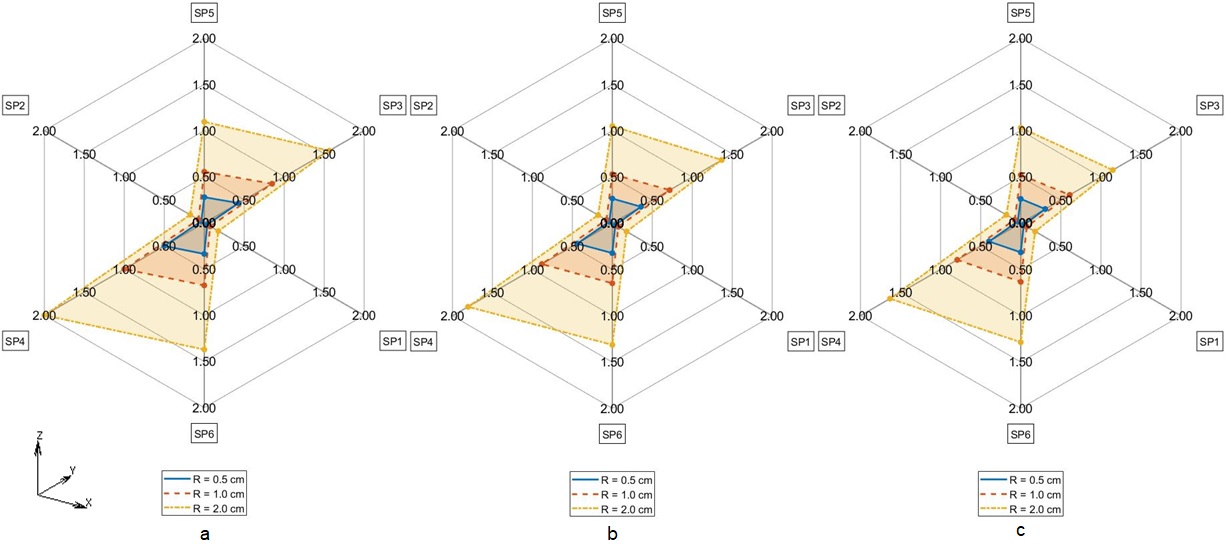


**Fig. 17:** TDE deviation in D_MAX_ for CMS for R = 0.5 cm (Solid line), R = 1.0 cm (Dashed line), R = 2 cm (Dash-dotted line) a. TA = 5° C, b. TA = 15° C, c. TA = 25° C


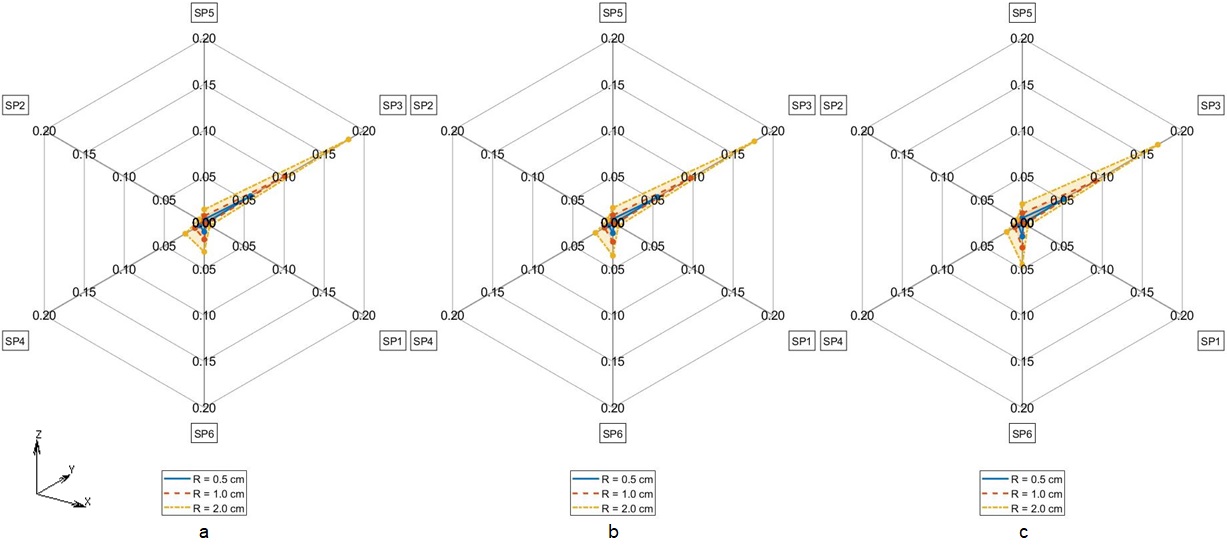


**Fig. 18:** TDE deviation in d_MAX_ for CMS for R = 0.5 cm (Solid line), R = 1.0 cm (Dashed line), R = 2 cm (Dash-dotted line) a. TA = 5° C, b. TA = 15° C, c. TA = 25° C


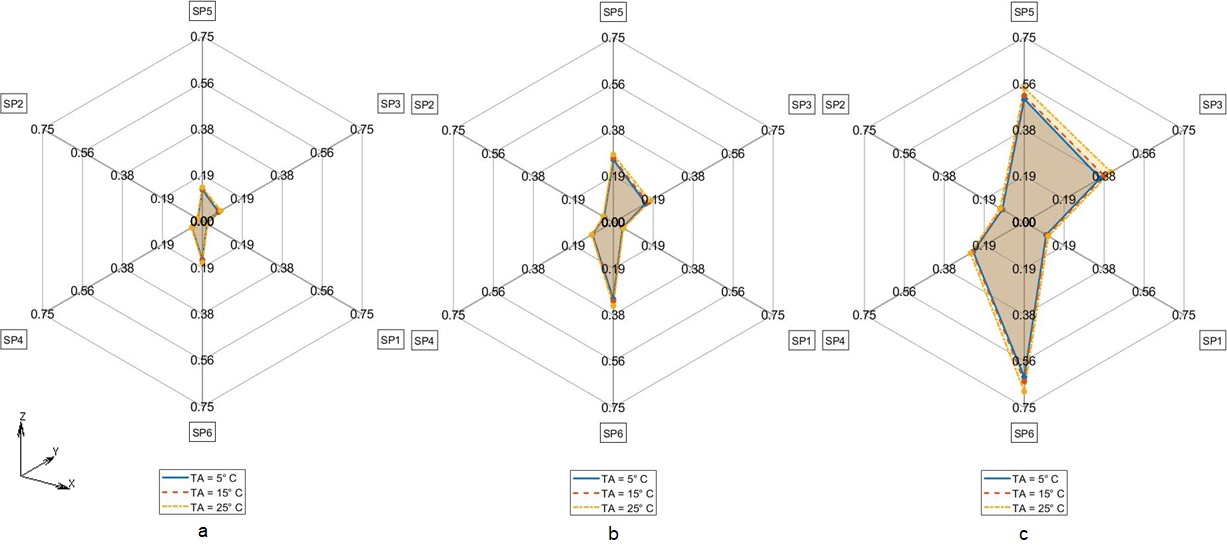


**Fig. 19:** TDE deviation in D_L2_ for CM for R = 0.5 cm (Solid line), R = 1.0 cm (Dashed line), R = 2 cm (Dash-dotted line) a. TA = 5° C, b. TA = 15° C, c. TA = 25° C


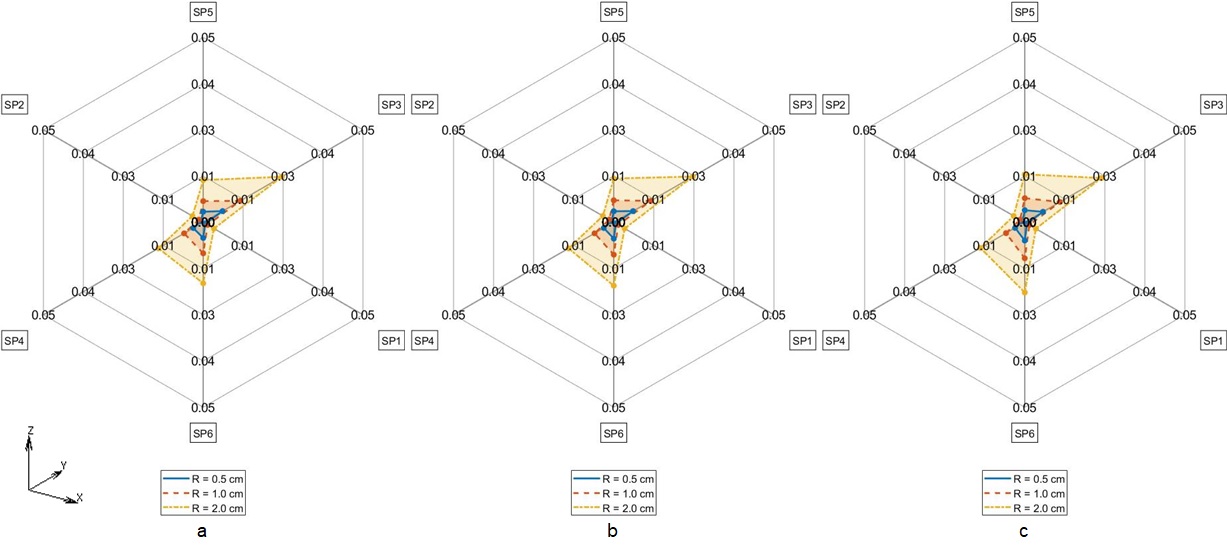


**Fig. 20:** TDE deviation in d_L2_ for CM for R = 0.5 cm (Solid line), R = 1.0 cm (Dashed line), R = 2 cm (Dash-dotted line) a. TA = 5° C, b. TA = 15° C, c. TA = 25° C


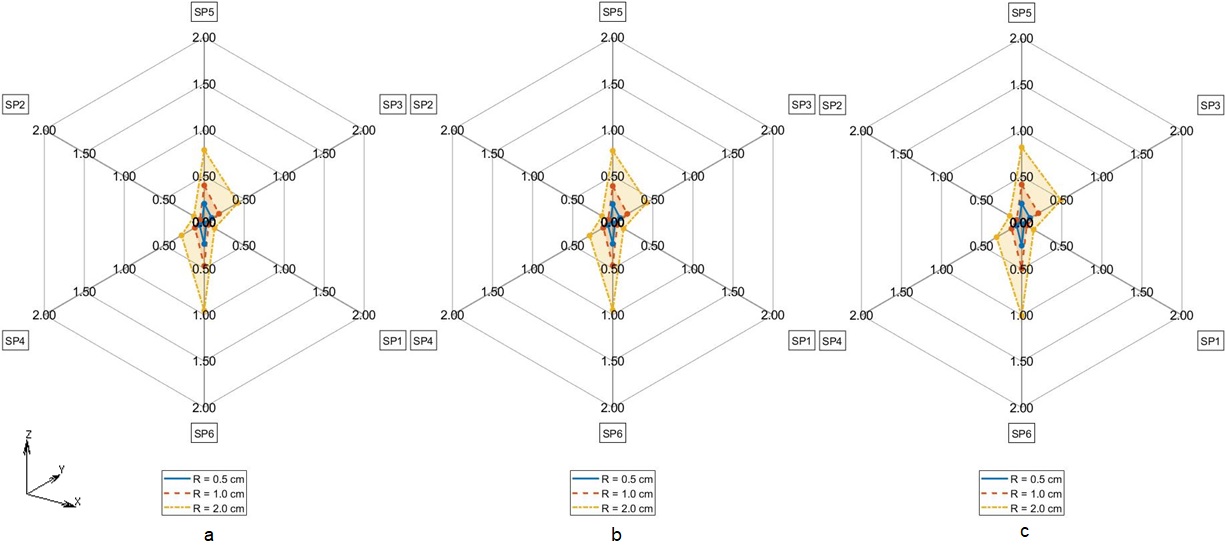


**Fig. 21:** TDE deviation in D_MAX_ for CM for R = 0.5 cm (Solid line), R = 1.0 cm (Dashed line), R = 2 cm (Dash-dotted line) a. TA = 5° C, b. TA = 15° C, c. TA = 25° C


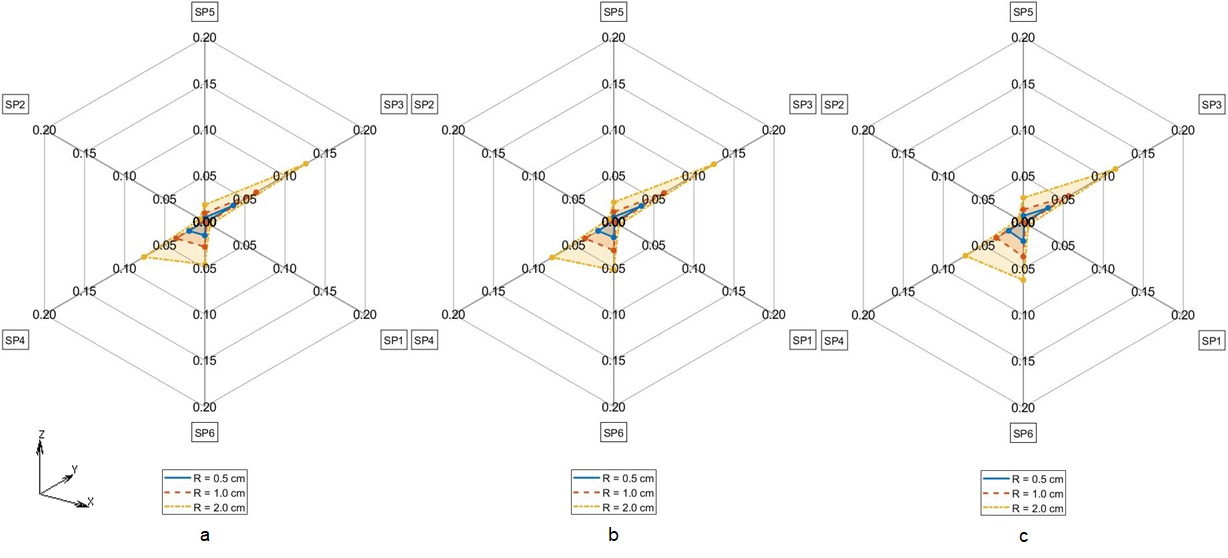


**Fig. 22:** TDE deviation in d_MAX_ for CM for R = 0.5 cm (Solid line), R = 1.0 cm (Dashed line), R = 2 cm (Dash-dotted line) a. TA = 5° C, b. TA = 15° C, c. TA = 25° C


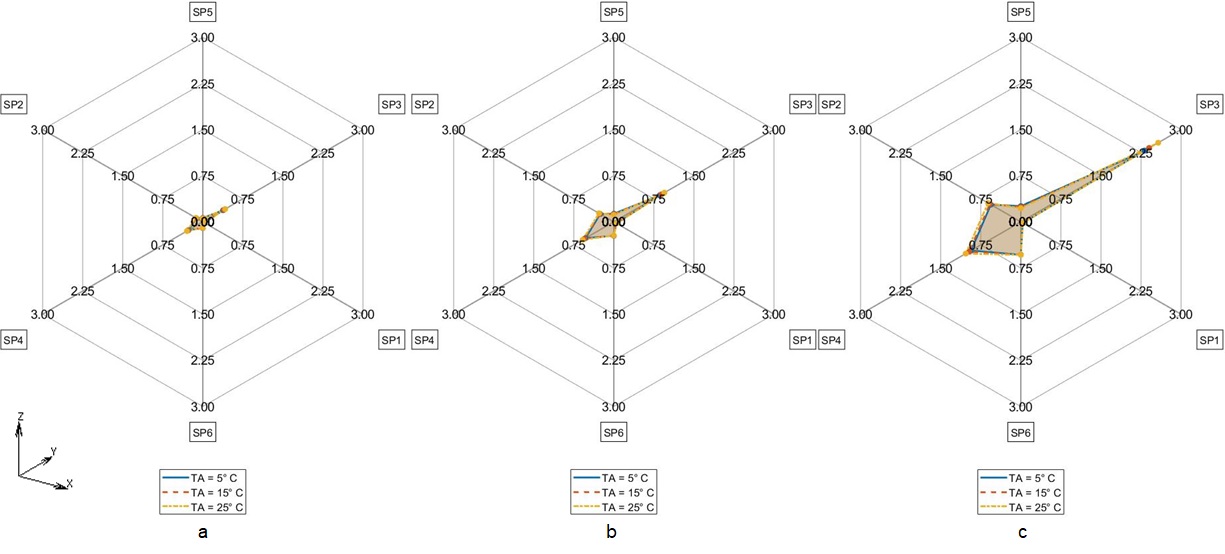


**Fig. 23:** TDE deviation in D_L2_ for CTM for TA = 5° C (Solid line), TA = 15° C (Dashed line), TA = 25° C (Dash-dotted line) a. R = 0.5 cm, b. R = 1 cm, c. R = 2 cm


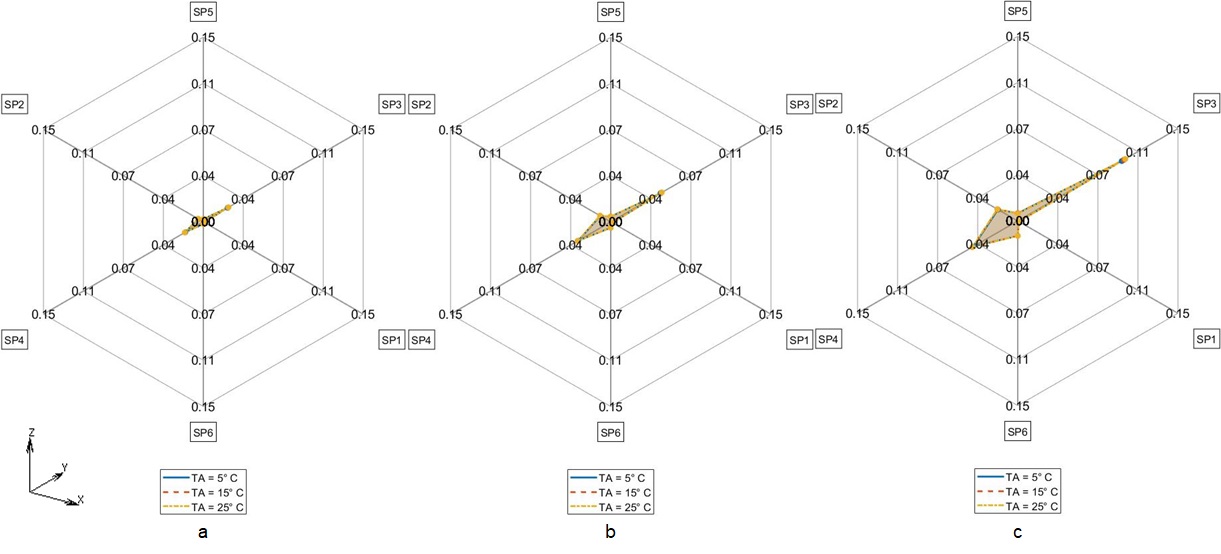


**Fig. 24:** TDE deviation in d_L2_ for CTM for TA = 5° C (Solid line), TA = 15° C (Dashed line), TA = 25° C (Dash-dotted line) a. R = 0.5 cm, b. R = 1 cm, c. R = 2 cm


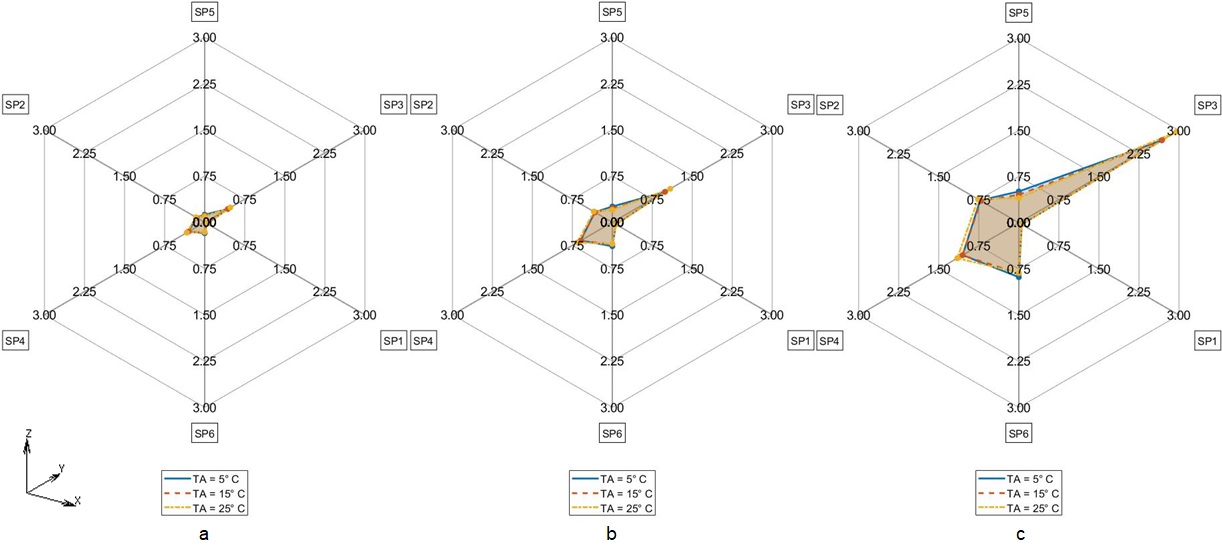


**Fig. 25:** TDE deviation in D_MAX_ for CTM for TA = 5° C (Solid line), TA = 15° C (Dashed line), TA = 25° C (Dash-dotted line) a. R = 0.5 cm, b. R = 1 cm, c. R = 2 cm


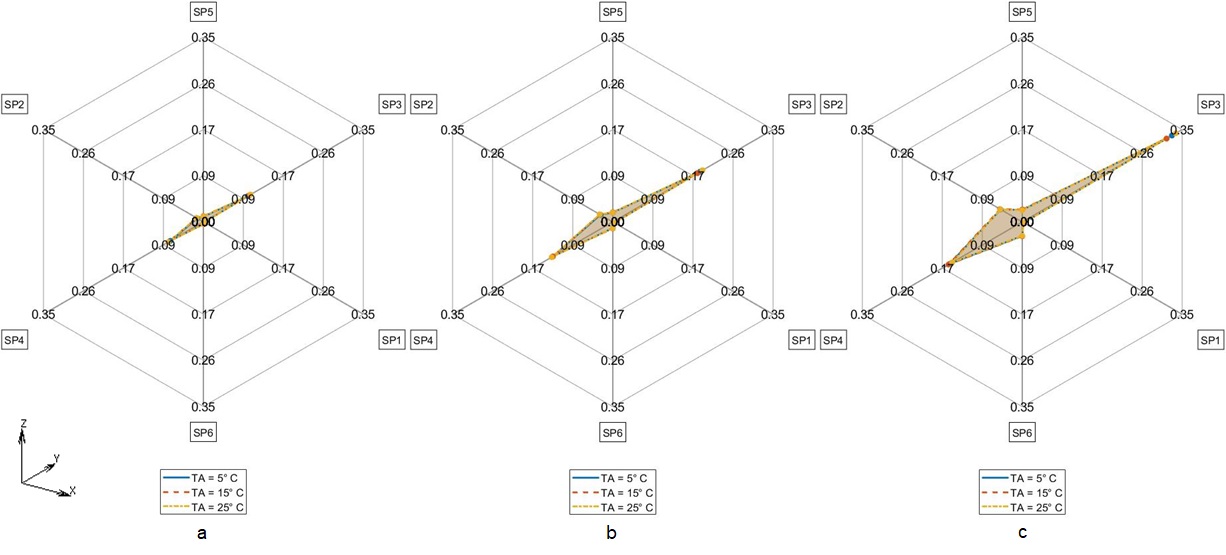


**Fig. 26:** TDE deviation in d_MAX_ for CTM for TA = 5° C (Solid line), TA = 15° C (Dashed line), TA = 25° C (Dash-dotted line) a. R = 0.5 cm, b. R = 1 cm, c. R = 2 cm


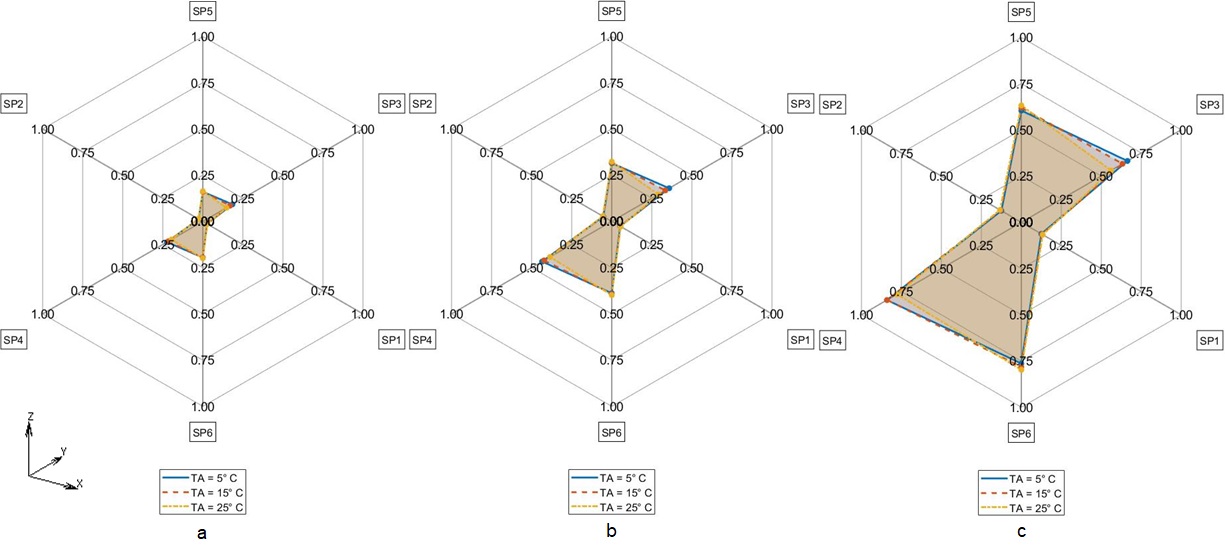


**Fig. 27:** TOD deviation in D_L2_ for CMS for TA = 5° C (Solid line), TA = 15° C (Dashed line), TA = 25° C (Dash-dotted line) a. R = 0.5 cm, b. R = 1 cm, c. R = 2 cm


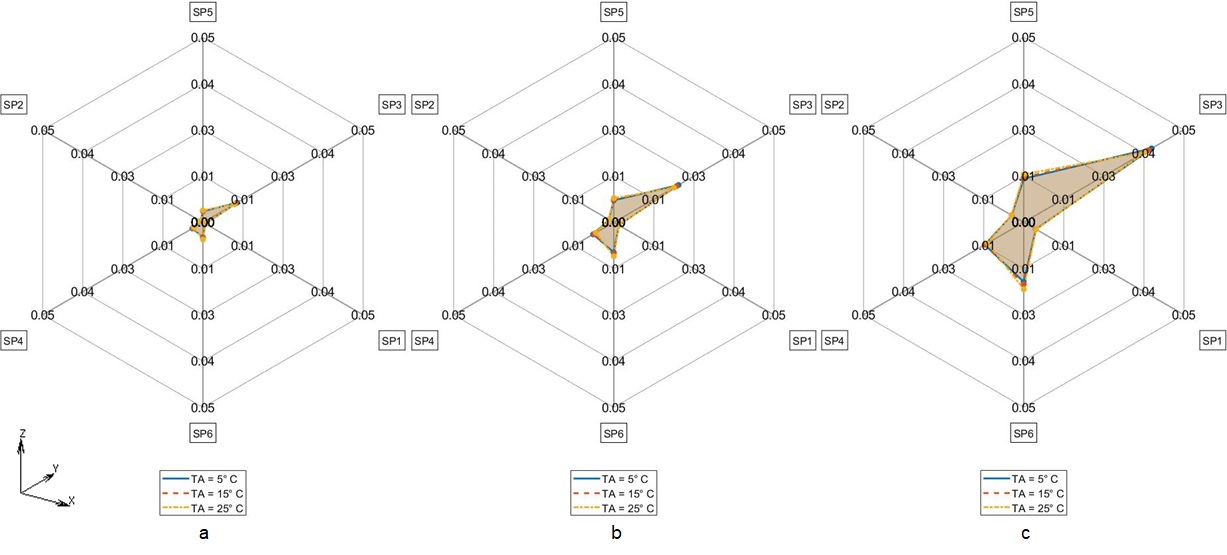


**Fig. 28:** TDE deviation in d_L2_ for CMS for TA = 5° C (Solid line), TA = 15° C (Dashed line), TA = 25° C (Dash-dotted line) a. R = 0.5 cm, b. R = 1 cm, c. R = 2 cm


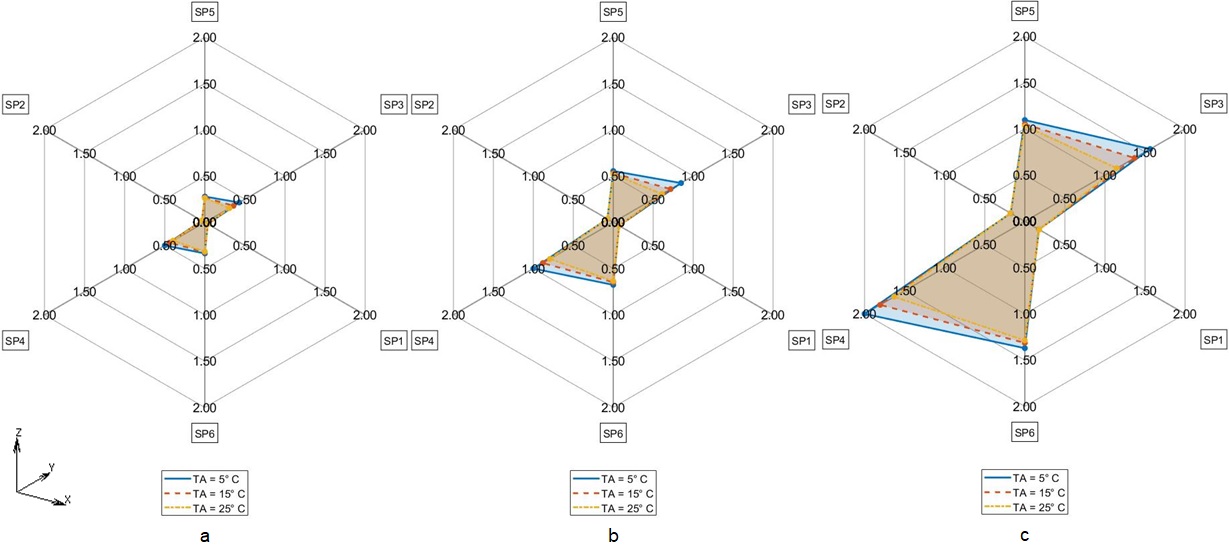
**Fig. 29:** TDE deviation in D_MAX_ for CMS for TA = 5° C (Solid line), TA = 15° C (Dashed line), TA = 25° C (Dash-dotted line) a. R = 0.5 cm, b. R = 1 cm, c. R = 2 cm


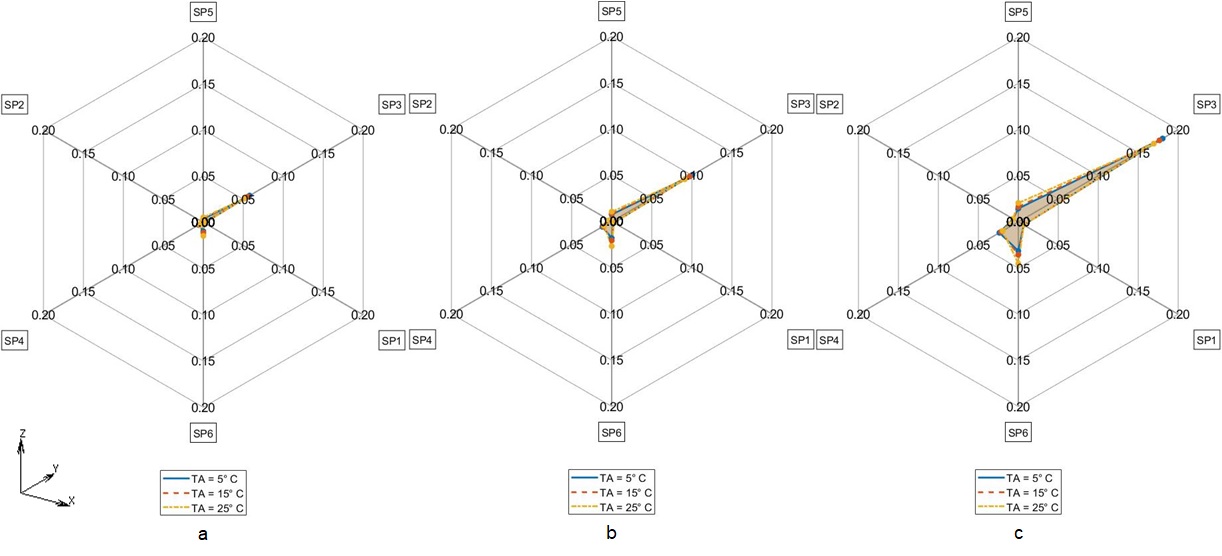


**Fig. 30:** TDE deviation in d_MAX_ for CMS for TA = 5° C (Solid line), TA = 15° C (Dashed line), TA = 25° C (Dash-dotted line) a. R = 0.5 cm, b. R = 1 cm, c. R = 2 cm


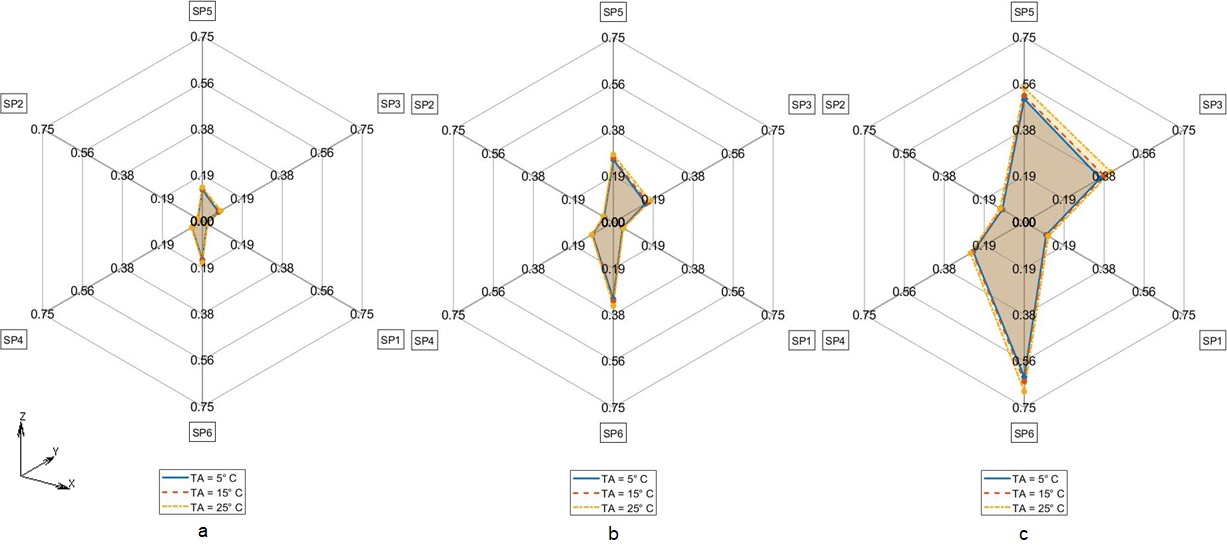


**Fig. 31:** TDE deviation in D_L2_ for CM for TA = 5° C (Solid line), TA = 15° C (Dashed line), TA = 25° C (Dash-dotted line) a. R = 0.5 cm, b. R = 1 cm, c. R = 2 cm


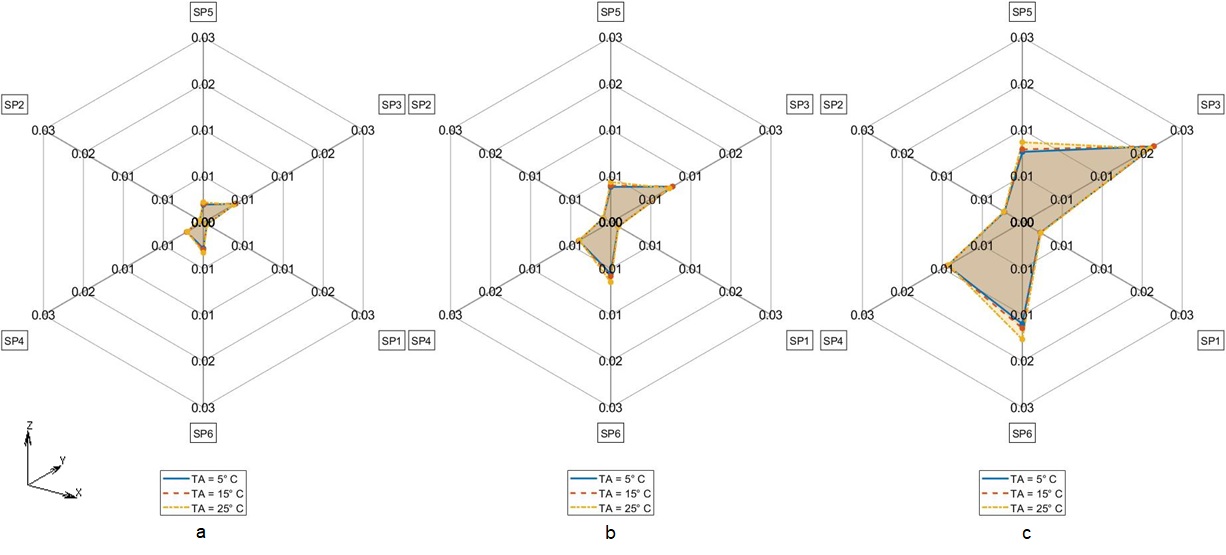


**Fig. 32:** TDE deviation in d_L2_ for CM for TA = 5° C (Solid line), TA = 15° C (Dashed line), TA = 25° C (Dash-dotted line) a. R = 0.5 cm, b. R = 1 cm, c. R = 2 cm


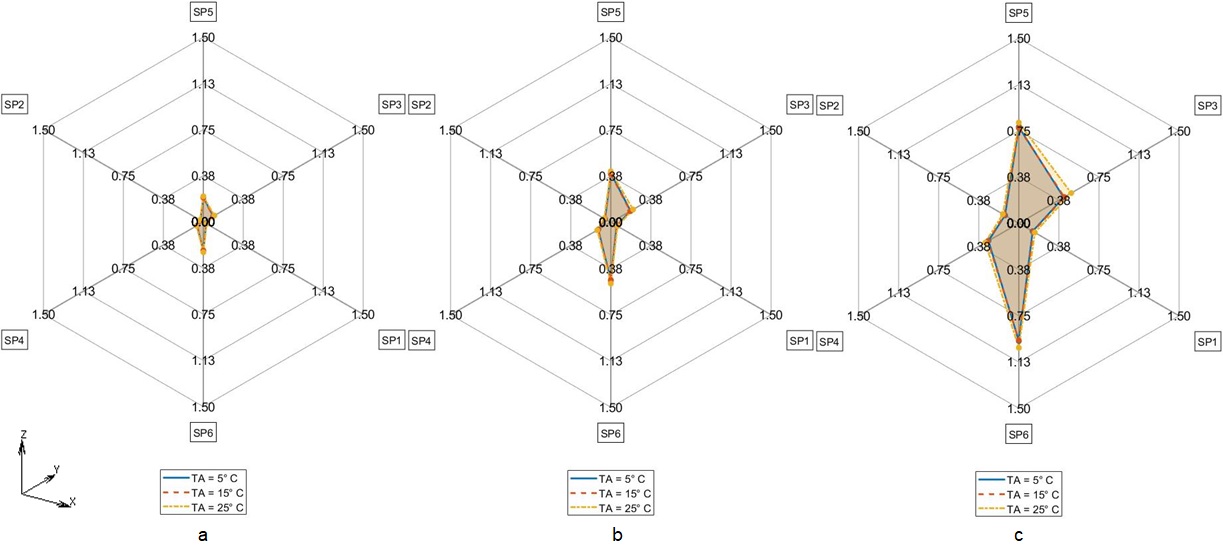


**Fig. 33:** TOD deviation in D_MAX_ for CM for TA = 5° C (Solid line), TA = 15° C (Dashed line), TA = 25° C (Dash-dotted line) a. R = 0.5 cm, b. R = 1 cm, c. R = 2 cm


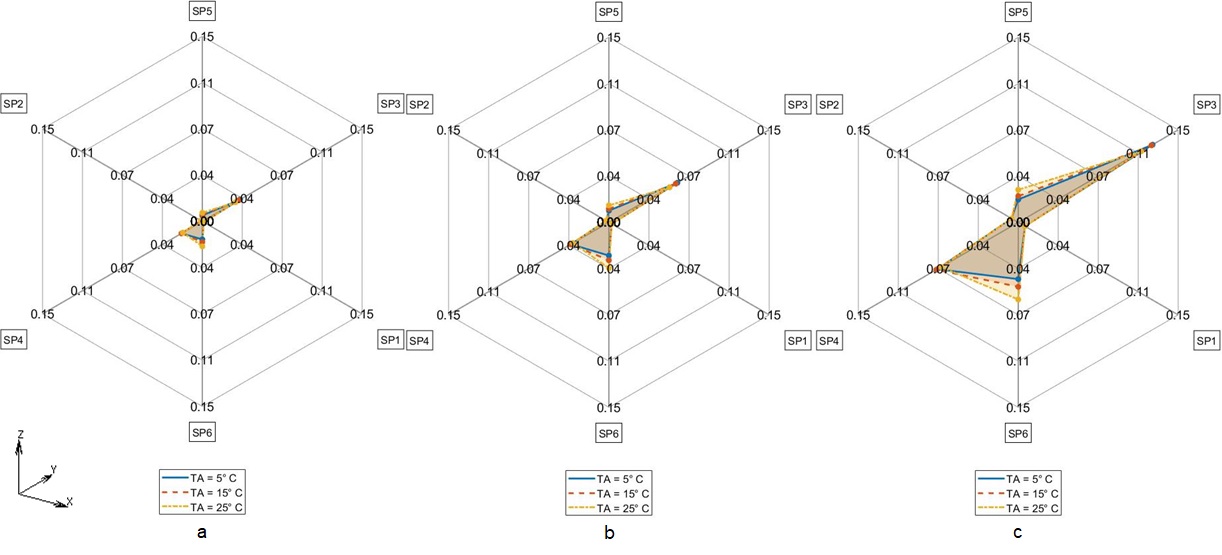


**Fig. 34:** TDE deviation in d_MAX_ for CM for TA = 5° C (Solid line), TA = 15° C (Dashed line), TA = 25° C (Dash-dotted line) a. R = 0.5 cm, b. R = 1 cm, c. R = 2 cm


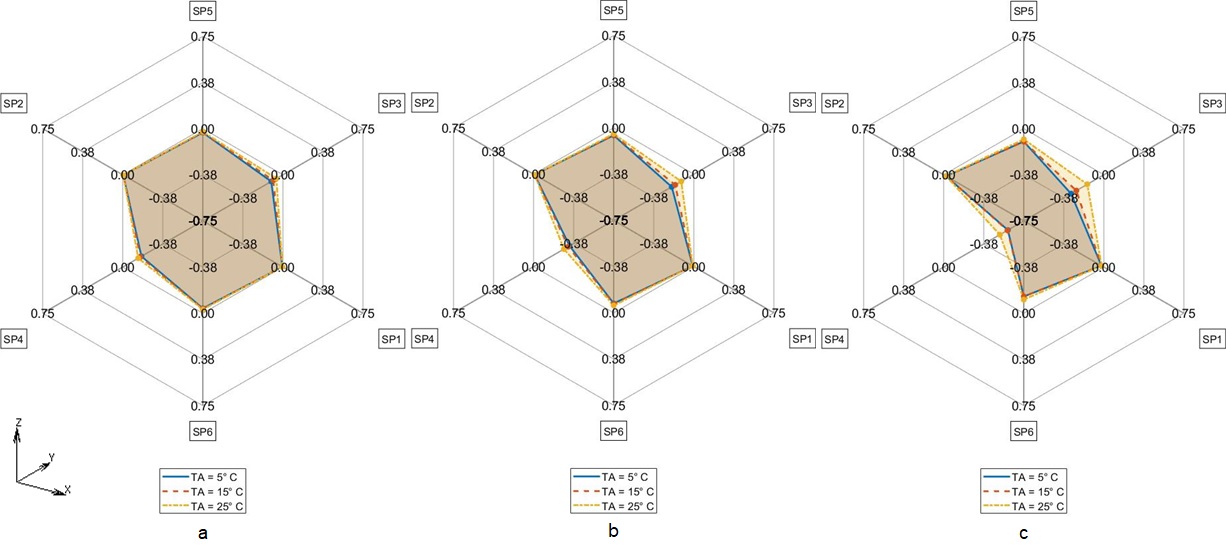


**Fig. 35:** Difference in TDE deviation in D_L2_ between CM vs CMS for TA = 5° C (Solid line), TA = 15° C (Dashed line), TA = 25° C (Dash-dotted line) a. R = 0.5 cm, b. R = 1 cm, c. R = 2 cm


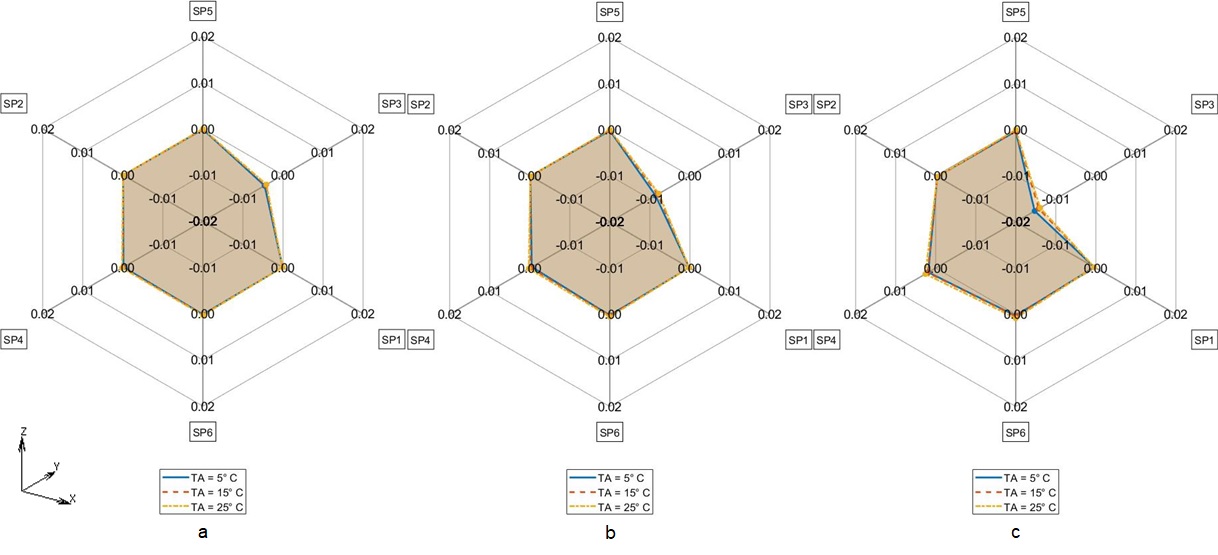


**Fig. 36:** Difference in TDE deviation in d_L2_ between CM vs CMS model for TA = 5° C (Solid line), TA = 15° C (Dashed line), TA = 25° C (Dash-dotted line) a. R = 0.5 cm, b. R = 1 cm, c. R = 2 cm


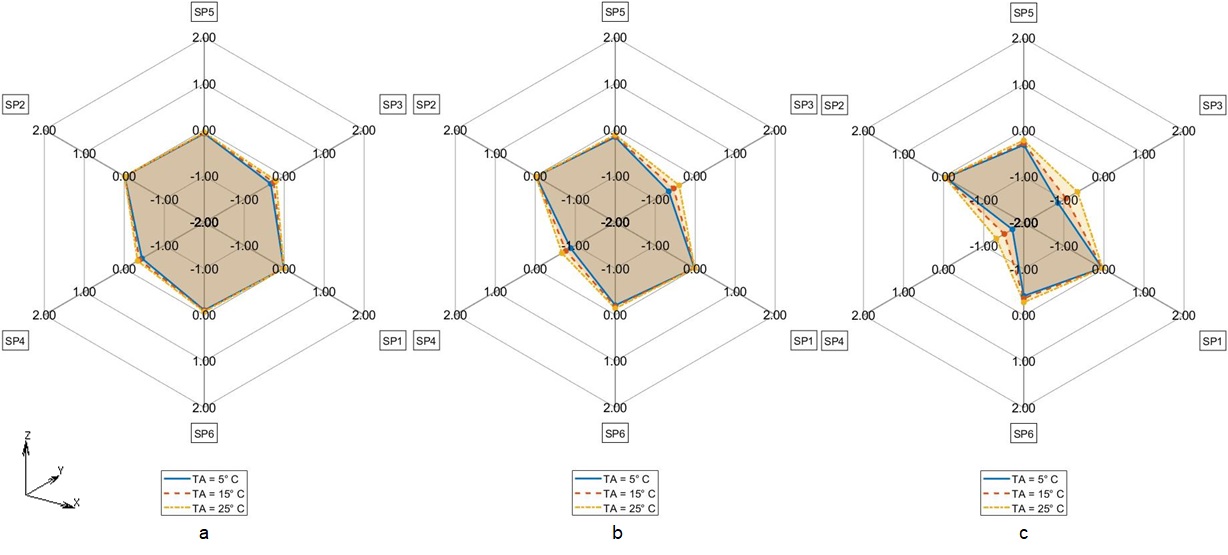


**Fig. 37:** Difference in TDE deviation in D_MAX_ between CM vs CMS model for TA = 5° C (Solid line), TA = 15° C (Dashed line), TA = 25° C (Dash-dotted line) a. R = 0.5 cm, b. R = 1 cm, c. R = 2 cm


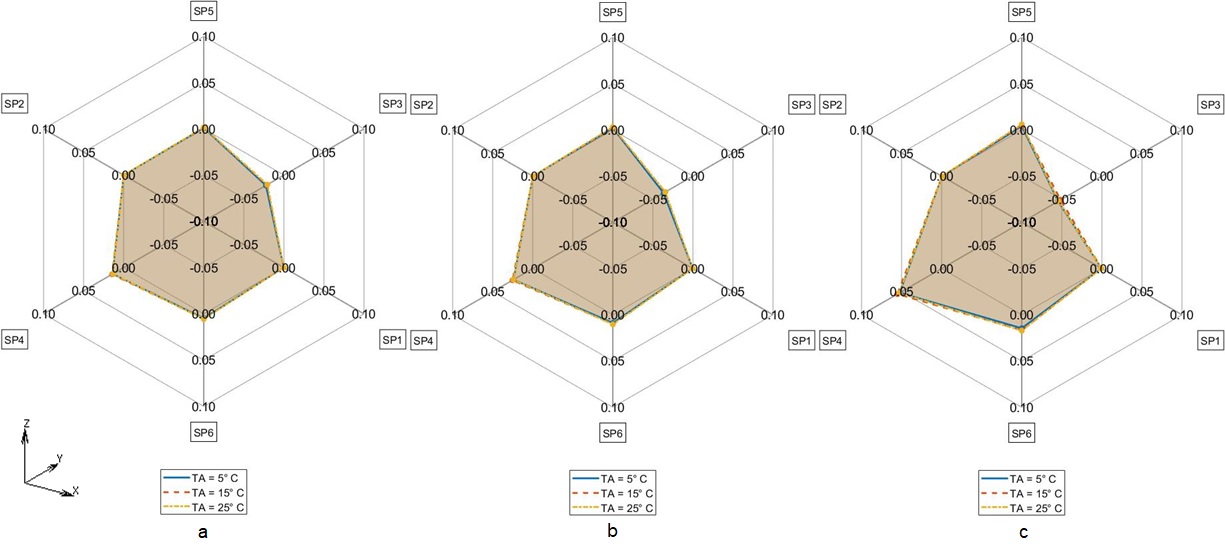


**Fig. 38:** Difference in TDE deviation in d_MAX_ between CM vs CMS for TA = 5° C (Solid line), TA = 15° C (Dashed line), TA = 25° C (Dash-dotted line) a. R = 0.5 cm, b. R = 1 cm, c. R = 2 cm


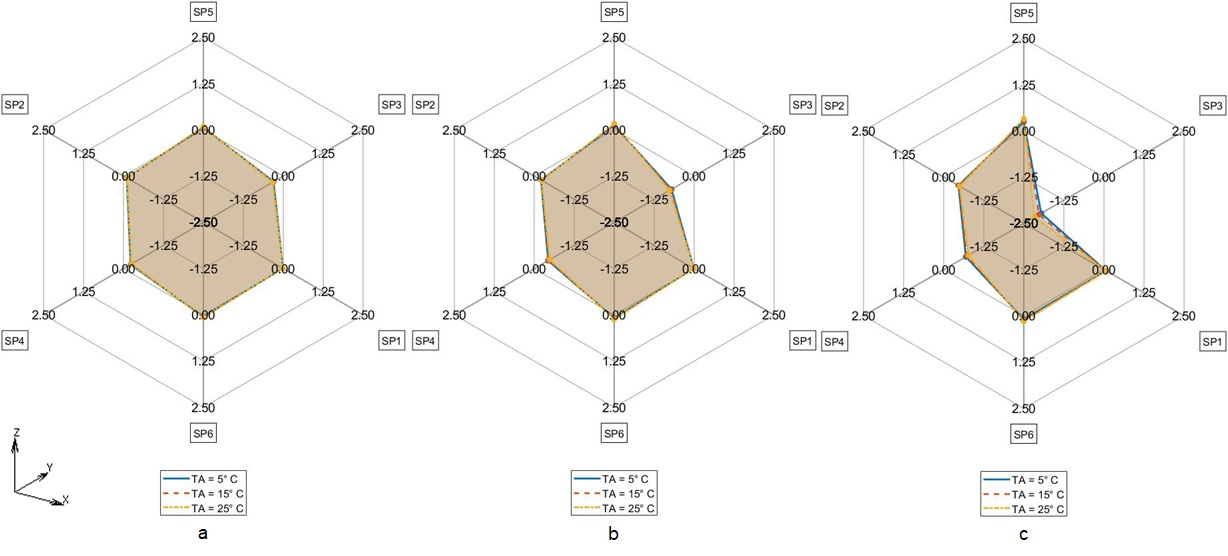


**Fig. 39:** Difference in TDE deviation in D_L2_ between CM vs CTM for TA = 5° C (Solid line), TA = 15° C (Dashed line), TA = 25° C (Dash-dotted line) a. R = 0.5 cm, b. R = 1 cm, c. R = 2 cm


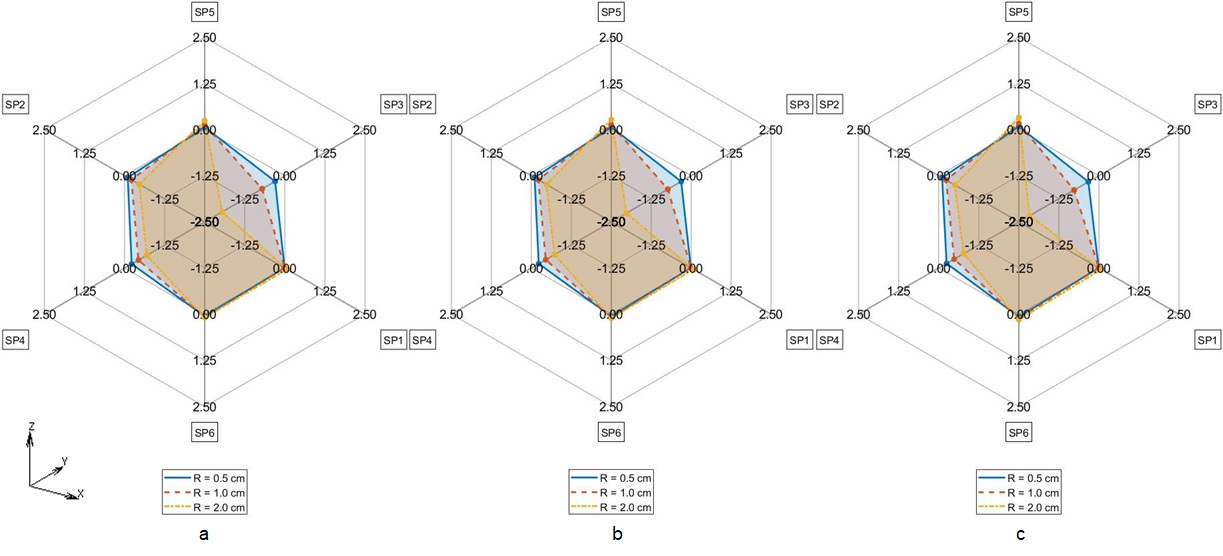


**Fig. 40:** Difference in TDE deviation in D_L2_ between CM vs CTM for R = 0.5 cm (Solid line), R = 1 cm (Dashed line), R = 2 cm (Dash-dotted line) a. TA = 5° C, b. TA = 15° C, c. TA = 25° C


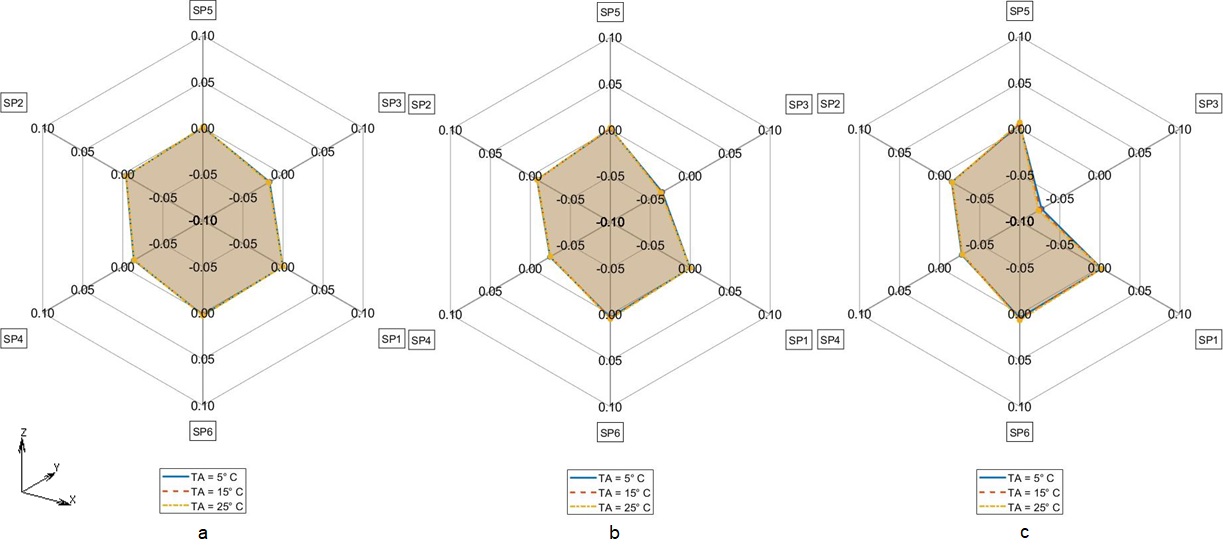


**Fig. 41:** Difference in TDE deviation in d_L2_ between CM vs CTM for TA = 5° C (Solid line), TA = 15° C (Dashed line), TA = 25° C (Dash-dotted line) a. R = 0.5 cm, b. R = 1 cm, c. R = 2 cm


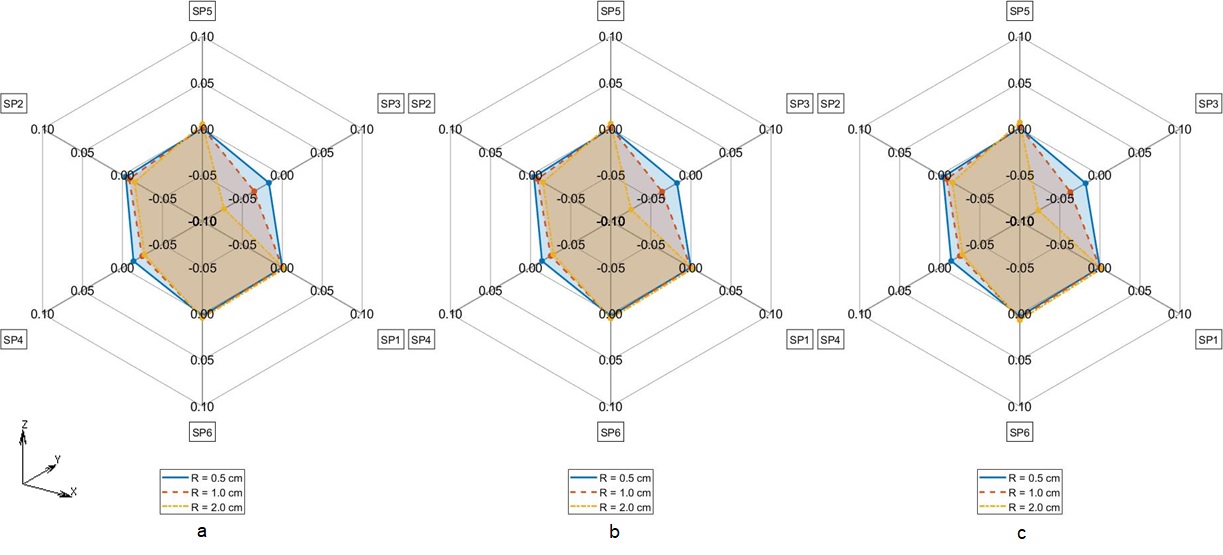


**Fig. 42:** Difference in TDE deviation in d_L2_ between CM vs CTM for R = 0.5 cm (Solid line), R = 1 cm (Dashed line), R = 2 cm (Dash-dotted line) a. TA = 5° C, b. TA = 15° C, c. TA = 25° C


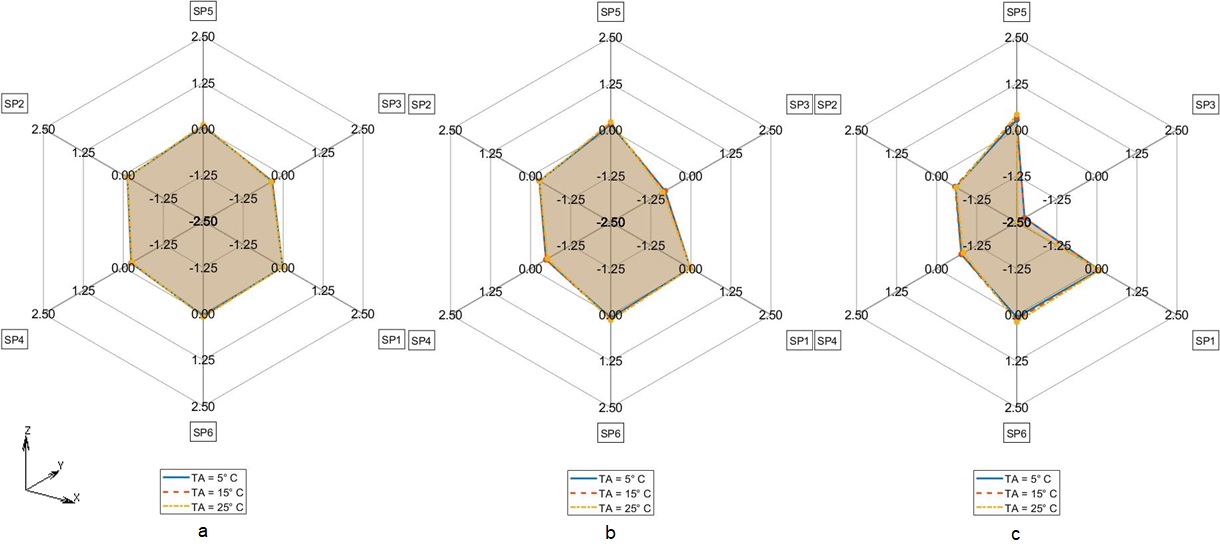


**Fig. 43:** Difference in TDE deviation in D_MAX_ between CM vs CTM for TA = 5° C (Solid line), TA = 15° C (Dashed line), TA = 25° C (Dash-dotted line) a. R = 0.5 cm, b. R = 1 cm, c. R = 2 cm


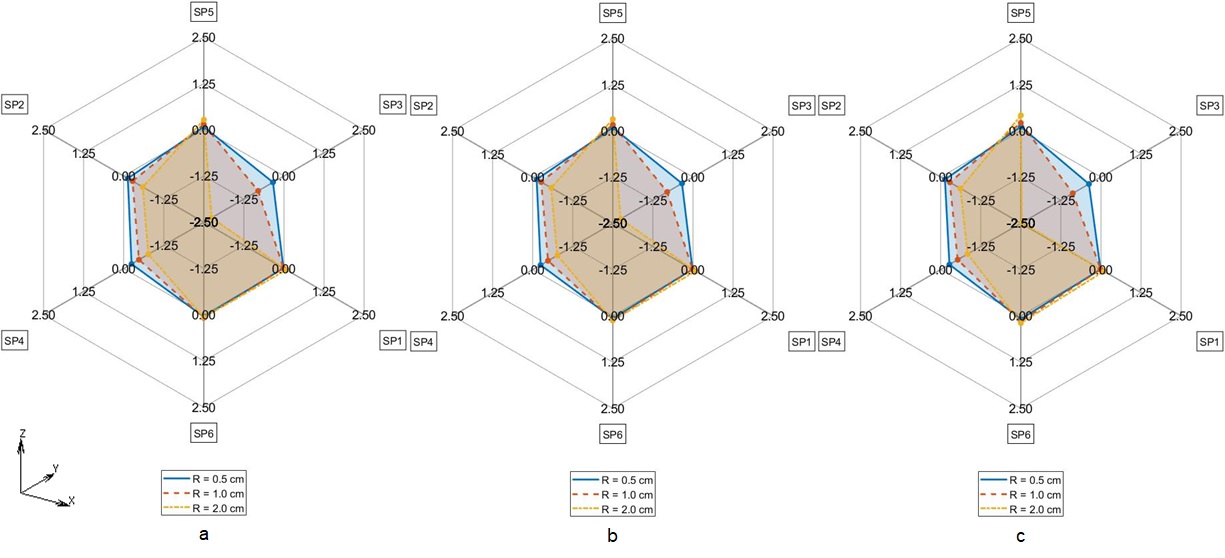


**Fig. 44:** Difference in TDE deviation in D_MAX_ between CM vs CTM for R = 0.5 cm (Solid line), R = 1 cm (Dashed line), R = 2 cm (Dash-dotted line) a. TA = 5° C, b. TA = 15° C, c. TA = 25° C


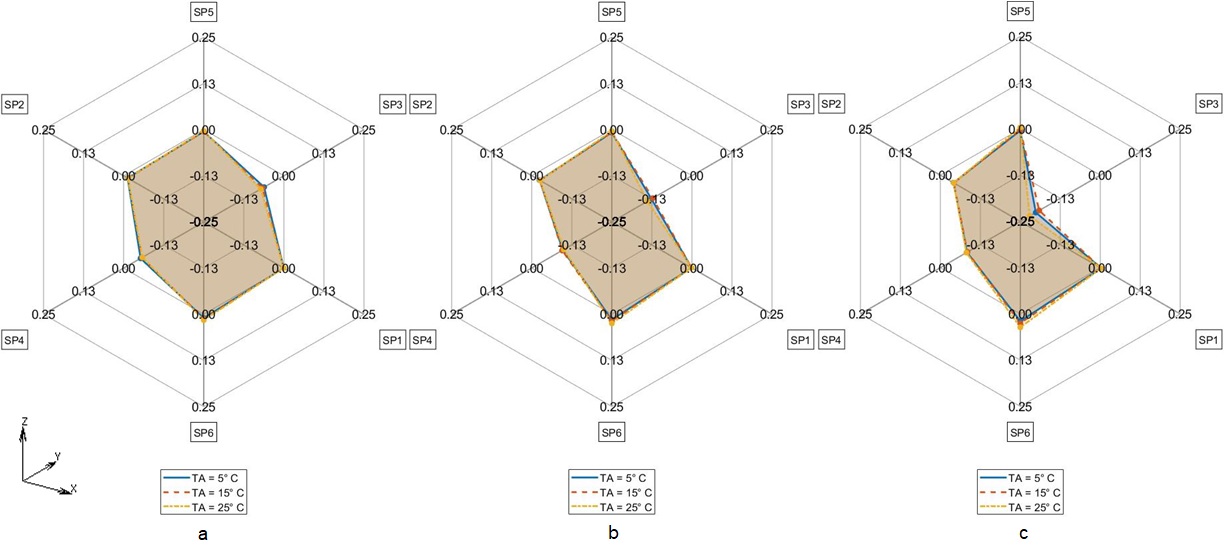


**Fig. 45:** Difference in TDE deviation in d_MAX_ between CM vs CTM for TA = 5° C (Solid line), TA = 15° C (Dashed line), TA = 25° C (Dash-dotted line) a. R = 0.5 cm, b. R = 1 cm, c. R = 2 cm


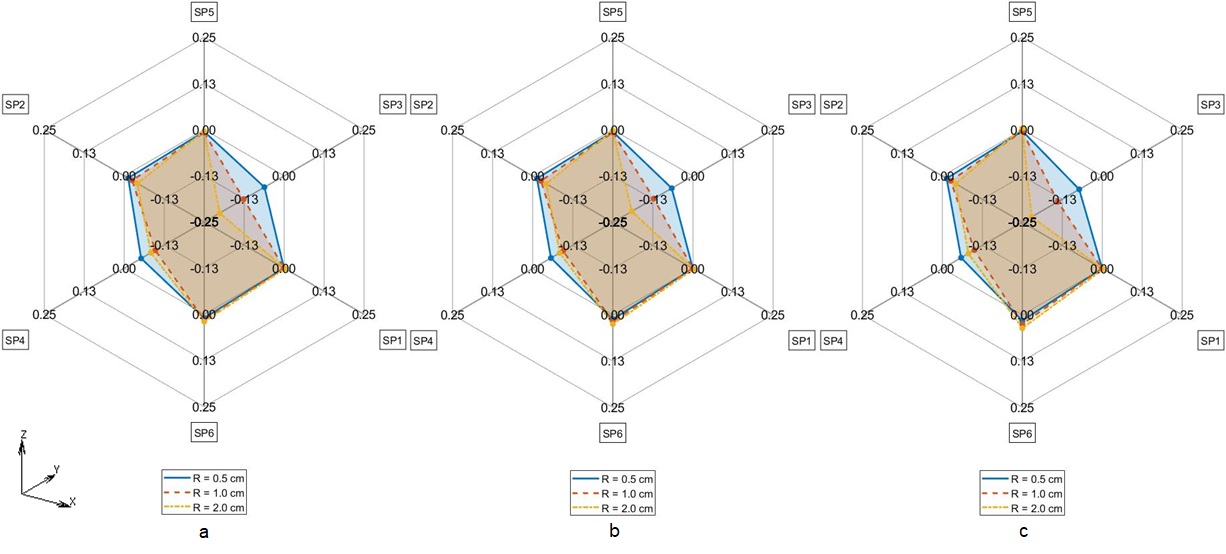


**Fig. 46:** Difference in TDE deviation in d_MAX_ between CM vs CTM for R = 0.5 cm (Solid line), R = 1 cm (Dashed line), R = 2 cm (Dash-dotted line) a. TA = 5° C, b. TA = 15° C, c. TA = 25° C

**Appendix**

| TDE deviation – CTM model - D_L2_ | | | | | | |
| --- | --- | --- | --- | --- | --- | --- |
| TA = 5° C, R = 0.5 cm | | | | | | |
|  | SP5 | SP3 | SP1 | SP6 | SP4 | SP2 |
| 0.5 ≤ Q < 1 | 0.033154 | 0.012357 | 0.011706 | 0.037054 | 0.384713 | 0.368970 |
| 0.5 < Q ≤ 0.3 | 0.361785 | 0.363103 | 0.016530 | 0.024081 | 0.026774 | 0.030003 |
| 0.3 < Q ≤ 0.2 | 0.018409 | 0.039704 | 0.059175 | 0.084013 | 0.283010 | 0.265253 |
| Q ≤ 0.1 | 0.258695 | 0.257514 | 0.089386 | 0.107827 | 0.112409 | 0.117968 |
| TA = 5° C, R = 1 cm | | | | | | |
| 0.5 ≤ Q < 1 | 0.071248 | 0.026240 | 0.024847 | 0.078675 | 0.872777 | 0.829095 |
| 0.5 < Q ≤ 0.3 | 0.816610 | 0.820621 | 0.030153 | 0.038886 | 0.042616 | 0.047560 |
| 0.3 < Q ≤ 0.2 | 0.064134 | 0.107450 | 0.146862 | 0.197328 | 0.561439 | 0.517443 |
| Q ≤ 0.1 | 0.505709 | 0.503591 | 0.191510 | 0.229326 | 0.239722 | 0.251558 |
| TA = 5° C, R = 2 cm | | | | | | |
| 0.5 ≤ Q < 1 | 0.123200 | 0.034041 | 0.071973 | 0.180640 | 2.311562 | 2.207778 |
| 0.5 < Q ≤ 0.3 | 2.189745 | 2.229798 | 0.023540 | 0.026183 | 0.029634 | 0.034980 |
| 0.3 < Q ≤ 0.2 | 0.221709 | 0.314866 | 0.395871 | 0.501582 | 0.984147 | 0.903881 |
| Q ≤ 0.1 | 0.887017 | 0.887690 | 0.433802 | 0.506385 | 0.529257 | 0.559350 |

**Tab. 14:** TDE deviation in CTM model at different RTML evaluated by D_L2_ at 5° C

| TDE deviation – CTM model - d_L2_ | | | | | | |
| --- | --- | --- | --- | --- | --- | --- |
| TA = 5° C, R = 0.5 cm | | | | | | |
|  | SP5 | SP3 | SP1 | SP6 | SP4 | SP2 |
| 0.5 ≤ Q < 1 | 0.004719 | 0.000583 | 0.000329 | 0.000793 | 0.049507 | 0.015795 |
| 0.5 < Q ≤ 0.3 | 0.010882 | 0.008168 | 0.001418 | 0.001010 | 0.000798 | 0.000668 |
| 0.3 < Q ≤ 0.2 | 0.001870 | 0.001621 | 0.001752 | 0.001851 | 0.036638 | 0.011229 |
| Q ≤ 0.1 | 0.007723 | 0.005763 | 0.009607 | 0.004572 | 0.003370 | 0.002643 |
| TA = 5° C, R = 1 cm | | | | | | |
| 0.5 ≤ Q < 1 | 0.008844 | 0.001195 | 0.000684 | 0.001653 | 0.099525 | 0.034441 |
| 0.5 < Q ≤ 0.3 | 0.024060 | 0.018165 | 0.002790 | 0.001583 | 0.001243 | 0.001041 |
| 0.3 < Q ≤ 0.2 | 0.006311 | 0.004302 | 0.004271 | 0.004294 | 0.065172 | 0.021177 |
| Q ≤ 0.1 | 0.014753 | 0.011073 | 0.018812 | 0.009439 | 0.007043 | 0.005551 |
| TA = 5° C, R = 2 cm | | | | | | |
| 0.5 ≤ Q < 1 | 0.011447 | 0.001410 | 0.001886 | 0.003621 | 0.194931 | 0.083597 |
| 0.5 < Q ≤ 0.3 | 0.060441 | 0.046882 | 0.001921 | 0.000972 | 0.000809 | 0.000726 |
| 0.3 < Q ≤ 0.2 | 0.017044 | 0.011651 | 0.010856 | 0.010449 | 0.084283 | 0.033861 |
| Q ≤ 0.1 | 0.024318 | 0.018595 | 0.033450 | 0.019064 | 0.014604 | 0.011756 |

**Tab. 15:** TDE deviation in CTM model at different RTML evaluated by d_L2_ at 5° C

| TDE deviation – CTM model - D_MAX_ | | | | | | |
| --- | --- | --- | --- | --- | --- | --- |
| TA = 5° C, R = 0.5 cm | | | | | | |
|  | SP5 | SP3 | SP1 | SP6 | SP4 | SP2 |
| 0.5 ≤ Q < 1 | 0.122852 | 0.093391 | 0.072357 | 0.055339 | 0.435910 | 0.386072 |
| 0.5 < Q ≤ 0.3 | 0.373923 | 0.369764 | 0.044560 | 0.038094 | 0.034864 | 0.032667 |
| 0.3 < Q ≤ 0.2 | 0.176460 | 0.141712 | 0.120125 | 0.103497 | 0.303744 | 0.270017 |
| Q ≤ 0.1 | 0.262275 | 0.260607 | 0.155169 | 0.134974 | 0.127751 | 0.123630 |
| TA = 5° C, R = 1 cm | | | | | | |
| 0.5 ≤ Q < 1 | 0.255475 | 0.191303 | 0.145863 | 0.109616 | 0.989255 | 0.875922 |
| 0.5 < Q ≤ 0.3 | 0.843773 | 0.833404 | 0.070145 | 0.059609 | 0.054310 | 0.050870 |
| 0.3 < Q ≤ 0.2 | 0.387430 | 0.311325 | 0.265372 | 0.231326 | 0.593254 | 0.527706 |
| Q ≤ 0.1 | 0.511466 | 0.507438 | 0.329621 | 0.286434 | 0.270129 | 0.261497 |
| TA = 5° C, R = 2 cm | | | | | | |
| 0.5 ≤ Q < 1 | 0.508475 | 0.368379 | 0.273719 | 0.200823 | 2.676332 | 2.381748 |
| 0.5 < Q ≤ 0.3 | 2.272005 | 2.236726 | 0.055936 | 0.045853 | 0.040050 | 0.036235 |
| 0.3 < Q ≤ 0.2 | 0.884038 | 0.707707 | 0.604823 | 0.533477 | 1.038491 | 0.925452 |
| Q ≤ 0.1 | 0.898327 | 0.891724 | 0.724933 | 0.628021 | 0.586598 | 0.566291 |

**Tab. 16:** TDE deviation in CTM model at different RTML evaluated by D_MAX_ at 5° C

| TDE deviation – CTM model - d_MAX_ | | | | | | |
| --- | --- | --- | --- | --- | --- | --- |
| TA = 5° C, R = 0.5 cm | | | | | | |
|  | SP5 | SP3 | SP1 | SP6 | SP4 | SP2 |
| 0.5 ≤ Q < 1 | 0.001465 | 0.001278 | 0.001158 | 0.001024 | 0.005271 | 0.005970 |
| 0.5 < Q ≤ 0.3 | 0.006818 | 0.009069 | 0.000538 | 0.000558 | 0.000604 | 0.000715 |
| 0.3 < Q ≤ 0.2 | 0.002112 | 0.001951 | 0.001921 | 0.001911 | 0.003698 | 0.004205 |
| Q ≤ 0.1 | 0.004827 | 0.006496 | 0.001871 | 0.002042 | 0.002282 | 0.002893 |
| TA = 5° C, R = 1 cm | | | | | | |
| 0.5 ≤ Q < 1 | 0.003046 | 0.002644 | 0.002363 | 0.002057 | 0.012074 | 0.013623 |
| 0.5 < Q ≤ 0.3 | 0.015561 | 0.020893 | 0.000846 | 0.000880 | 0.000954 | 0.001144 |
| 0.3 < Q ≤ 0.2 | 0.004641 | 0.004335 | 0.004339 | 0.004333 | 0.007308 | 0.008287 |
| Q ≤ 0.1 | 0.009527 | 0.013001 | 0.003975 | 0.004364 | 0.004884 | 0.006252 |
| TA = 5° C, R = 2 cm | | | | | | |
| 0.5 ≤ Q < 1 | 0.006064 | 0.005222 | 0.004572 | 0.003907 | 0.033724 | 0.037836 |
| 0.5 < Q ≤ 0.3 | 0.043327 | 0.059564 | 0.000673 | 0.000667 | 0.000702 | 0.000809 |
| 0.3 < Q ≤ 0.2 | 0.010602 | 0.010100 | 0.010326 | 0.010832 | 0.013126 | 0.014846 |
| Q ≤ 0.1 | 0.017141 | 0.024139 | 0.008870 | 0.009730 | 0.010910 | 0.014342 |

**Tab. 17:** TDE deviation in CTM model at different RTML evaluated by d_MAX_ at 5° C

| TDE deviation – CTM model - D_L2_ | | | | | | |
| --- | --- | --- | --- | --- | --- | --- |
| TA = 15° C, R = 0.5 cm | | | | | | |
|  | SP5 | SP3 | SP1 | SP6 | SP4 | SP2 |
| 0.5 ≤ Q < 1 | 0.034000 | 0.014584 | 0.008373 | 0.032115 | 0.387431 | 0.376427 |
| 0.5 < Q ≤ 0.3 | 0.373081 | 0.374986 | 0.016424 | 0.024162 | 0.027165 | 0.030259 |
| 0.3 < Q ≤ 0.2 | 0.017653 | 0.037735 | 0.056709 | 0.080530 | 0.285213 | 0.270456 |
| Q ≤ 0.1 | 0.267834 | 0.266388 | 0.089392 | 0.109107 | 0.114875 | 0.120656 |
| TA = 15° C, R = 1 cm | | | | | | |
| 0.5 ≤ Q < 1 | 0.073232 | 0.030952 | 0.018210 | 0.068358 | 0.880669 | 0.843766 |
| 0.5 < Q ≤ 0.3 | 0.847183 | 0.846676 | 0.030203 | 0.038963 | 0.043699 | 0.047914 |
| 0.3 < Q ≤ 0.2 | 0.062824 | 0.103482 | 0.143903 | 0.190964 | 0.566447 | 0.526371 |
| Q ≤ 0.1 | 0.527061 | 0.519949 | 0.192018 | 0.231170 | 0.246890 | 0.256953 |
| TA = 15° C, R = 2 cm | | | | | | |
| 0.5 ≤ Q < 1 | 0.127492 | 0.042330 | 0.058405 | 0.163042 | 2.336154 | 2.264867 |
| 0.5 < Q ≤ 0.3 | 2.279777 | 2.304328 | 0.023712 | 0.026848 | 0.030154 | 0.035107 |
| 0.3 < Q ≤ 0.2 | 0.217589 | 0.314012 | 0.394301 | 0.492990 | 0.991322 | 0.936006 |
| Q ≤ 0.1 | 0.920564 | 0.918638 | 0.432890 | 0.519105 | 0.546082 | 0.571961 |

**Tab. 18:** TDE deviation in CTM model at different RTML evaluated by D_L2_ at 15° C

| TDE deviation – CTM model - d_L2_ | | | | | | |
| --- | --- | --- | --- | --- | --- | --- |
| TA = 15° C, R = 0.5 cm | | | | | | |
|  | SP5 | SP3 | SP1 | SP6 | SP4 | SP2 |
| 0.5 ≤ Q < 1 | 0.004911 | 0.000695 | 0.000238 | 0.000699 | 0.051116 | 0.016548 |
| 0.5 < Q ≤ 0.3 | 0.011517 | 0.008653 | 0.001440 | 0.001041 | 0.000831 | 0.000691 |
| 0.3 < Q ≤ 0.2 | 0.001899 | 0.001584 | 0.001723 | 0.001817 | 0.037823 | 0.011754 |
| Q ≤ 0.1 | 0.008205 | 0.006109 | 0.009857 | 0.004751 | 0.003534 | 0.002773 |
| TA = 15° C, R = 1 cm | | | | | | |
| 0.5 ≤ Q < 1 | 0.009154 | 0.001420 | 0.000505 | 0.001459 | 0.102076 | 0.035964 |
| 0.5 < Q ≤ 0.3 | 0.025585 | 0.019173 | 0.002849 | 0.001627 | 0.001305 | 0.001072 |
| 0.3 < Q ≤ 0.2 | 0.006382 | 0.004257 | 0.004286 | 0.004246 | 0.066813 | 0.022076 |
| Q ≤ 0.1 | 0.015741 | 0.011675 | 0.019245 | 0.009762 | 0.007435 | 0.005800 |
| TA = 15° C, R = 2 cm | | | | | | |
| 0.5 ≤ Q < 1 | 0.011886 | 0.001803 | 0.001545 | 0.003303 | 0.199597 | 0.087672 |
| 0.5 < Q ≤ 0.3 | 0.064017 | 0.049174 | 0.001972 | 0.001021 | 0.000836 | 0.000738 |
| 0.3 < Q ≤ 0.2 | 0.017142 | 0.011883 | 0.010971 | 0.010411 | 0.086249 | 0.035845 |
| Q ≤ 0.1 | 0.025611 | 0.019500 | 0.034064 | 0.019992 | 0.015295 | 0.012189 |

**Tab. 19:** TDE deviation in CTM model at different RTML evaluated by d_L2_ at 15° C

| TDE deviation – CTM model - D_MAX_ | | | | | | |
| --- | --- | --- | --- | --- | --- | --- |
| TA = 15° C, R = 0.5 cm | | | | | | |
|  | SP5 | SP3 | SP1 | SP6 | SP4 | SP2 |
| 0.5 ≤ Q < 1 | 0.109587 | 0.092683 | 0.070249 | 0.055203 | 0.433453 | 0.421614 |
| 0.5 < Q ≤ 0.3 | 0.396629 | 0.397572 | 0.043044 | 0.040533 | 0.036175 | 0.034646 |
| 0.3 < Q ≤ 0.2 | 0.162133 | 0.145500 | 0.120709 | 0.106719 | 0.303075 | 0.295050 |
| Q ≤ 0.1 | 0.280676 | 0.280975 | 0.151627 | 0.145669 | 0.133883 | 0.131968 |
| TA = 15° C, R = 1 cm | | | | | | |
| 0.5 ≤ Q < 1 | 0.227928 | 0.188492 | 0.141034 | 0.109312 | 0.984172 | 0.952563 |
| 0.5 < Q ≤ 0.3 | 0.898900 | 0.898802 | 0.067610 | 0.063170 | 0.056295 | 0.054221 |
| 0.3 < Q ≤ 0.2 | 0.357879 | 0.319596 | 0.267523 | 0.240491 | 0.592053 | 0.573809 |
| Q ≤ 0.1 | 0.548922 | 0.548305 | 0.322320 | 0.308006 | 0.282986 | 0.280491 |
| TA = 15° C, R = 2 cm | | | | | | |
| 0.5 ≤ Q < 1 | 0.454763 | 0.360672 | 0.264323 | 0.197095 | 2.684084 | 2.569004 |
| 0.5 < Q ≤ 0.3 | 2.423443 | 2.368025 | 0.053276 | 0.047718 | 0.041156 | 0.037929 |
| 0.3 < Q ≤ 0.2 | 0.823447 | 0.725776 | 0.612409 | 0.552633 | 1.038416 | 0.998494 |
| Q ≤ 0.1 | 0.963543 | 0.969501 | 0.709299 | 0.669590 | 0.617098 | 0.602305 |

**Tab. 20:** TDE deviation in CTM model at different RTML evaluated by D_MAX_ at 15° C

| TDE deviation – CTM model - d_MAX_ | | | | | | |
| --- | --- | --- | --- | --- | --- | --- |
| TA = 15° C, R = 0.5 cm | | | | | | |
|  | SP5 | SP3 | SP1 | SP6 | SP4 | SP2 |
| 0.5 ≤ Q < 1 | 0.001318 | 0.001262 | 0.001114 | 0.001008 | 0.005732 | 0.006256 |
| 0.5 < Q ≤ 0.3 | 0.007221 | 0.008891 | 0.000552 | 0.000574 | 0.000633 | 0.000711 |
| 0.3 < Q ≤ 0.2 | 0.001995 | 0.001981 | 0.001946 | 0.001951 | 0.004026 | 0.004412 |
| Q ≤ 0.1 | 0.005134 | 0.006412 | 0.001982 | 0.002120 | 0.002402 | 0.002837 |
| TA = 15° C, R = 1 cm | | | | | | |
| 0.5 ≤ Q < 1 | 0.002739 | 0.002596 | 0.002266 | 0.002030 | 0.013085 | 0.014271 |
| 0.5 < Q ≤ 0.3 | 0.016606 | 0.021194 | 0.000870 | 0.000904 | 0.001006 | 0.001162 |
| 0.3 < Q ≤ 0.2 | 0.004422 | 0.004399 | 0.004448 | 0.004448 | 0.007922 | 0.008693 |
| Q ≤ 0.1 | 0.010199 | 0.013373 | 0.004234 | 0.004532 | 0.005183 | 0.006293 |
| TA = 15° C, R = 2 cm | | | | | | |
| 0.5 ≤ Q < 1 | 0.005466 | 0.005094 | 0.004388 | 0.003804 | 0.036244 | 0.039764 |
| 0.5 < Q ≤ 0.3 | 0.045489 | 0.062401 | 0.000674 | 0.000682 | 0.000735 | 0.000867 |
| 0.3 < Q ≤ 0.2 | 0.010240 | 0.010230 | 0.010672 | 0.011491 | 0.014115 | 0.015607 |
| Q ≤ 0.1 | 0.018121 | 0.025836 | 0.009420 | 0.010124 | 0.011521 | 0.015418 |

**Tab. 21:** TDE deviation in CTM model at different RTML evaluated by d_MAX_ at 15° C

| TDE deviation – CTM model - D_L2_ | | | | | | |
| --- | --- | --- | --- | --- | --- | --- |
| TA = 25° C, R = 0.5 cm | | | | | | |
|  | SP5 | SP3 | SP1 | SP6 | SP4 | SP2 |
| 0.5 ≤ Q < 1 | 0.034536 | 0.016970 | 0.005564 | 0.028061 | 0.395109 | 0.393431 |
| 0.5 < Q ≤ 0.3 | 0.392303 | 0.404684 | 0.016654 | 0.024832 | 0.027922 | 0.031937 |
| 0.3 < Q ≤ 0.2 | 0.017734 | 0.037136 | 0.055208 | 0.080305 | 0.290139 | 0.283859 |
| Q ≤ 0.1 | 0.280957 | 0.288119 | 0.090953 | 0.112845 | 0.119322 | 0.128715 |
| TA = 25° C, R = 1 cm | | | | | | |
| 0.5 ≤ Q < 1 | 0.074461 | 0.036131 | 0.011855 | 0.059509 | 0.898597 | 0.886643 |
| 0.5 < Q ≤ 0.3 | 0.886077 | 0.917201 | 0.030809 | 0.040398 | 0.044659 | 0.050790 |
| 0.3 < Q ≤ 0.2 | 0.063581 | 0.104024 | 0.140865 | 0.193096 | 0.575938 | 0.553573 |
| Q ≤ 0.1 | 0.549049 | 0.565136 | 0.195609 | 0.240165 | 0.254490 | 0.275251 |
| TA = 25° C, R = 2 cm | | | | | | |
| 0.5 ≤ Q < 1 | 0.129937 | 0.048794 | 0.043252 | 0.144853 | 2.407056 | 2.378557 |
| 0.5 < Q ≤ 0.3 | 2.395564 | 2.507626 | 0.024639 | 0.027686 | 0.031014 | 0.036897 |
| 0.3 < Q ≤ 0.2 | 0.223995 | 0.314890 | 0.392587 | 0.505402 | 1.014086 | 0.974188 |
| Q ≤ 0.1 | 0.966186 | 1.001202 | 0.446445 | 0.532950 | 0.564899 | 0.615222 |

**Tab. 22:** TDE deviation in CTM model at different RTML evaluated by D_L2_ at 25° C

| TDE deviation – CTM model - d_L2_ | | | | | | |
| --- | --- | --- | --- | --- | --- | --- |
| TA = 25° C, R = 0.5 cm | | | | | | |
|  | SP5 | SP3 | SP1 | SP6 | SP4 | SP2 |
| 0.5 ≤ Q < 1 | 0.004864 | 0.000804 | 0.000161 | 0.000601 | 0.051506 | 0.017379 |
| 0.5 < Q ≤ 0.3 | 0.012108 | 0.009229 | 0.001465 | 0.001075 | 0.000854 | 0.000721 |
| 0.3 < Q ≤ 0.2 | 0.001914 | 0.001566 | 0.001677 | 0.001793 | 0.037950 | 0.012403 |
| Q ≤ 0.1 | 0.008607 | 0.006537 | 0.010024 | 0.004937 | 0.003671 | 0.002925 |
| TA = 25° C, R = 1 cm | | | | | | |
| 0.5 ≤ Q < 1 | 0.009042 | 0.001644 | 0.000334 | 0.001249 | 0.102253 | 0.037794 |
| 0.5 < Q ≤ 0.3 | 0.026679 | 0.020519 | 0.002897 | 0.001688 | 0.001331 | 0.001124 |
| 0.3 < Q ≤ 0.2 | 0.006409 | 0.004281 | 0.004187 | 0.004246 | 0.066312 | 0.023255 |
| Q ≤ 0.1 | 0.016368 | 0.012557 | 0.019433 | 0.010144 | 0.007642 | 0.006140 |
| TA = 25° C, R = 2 cm | | | | | | |
| 0.5 ≤ Q < 1 | 0.011621 | 0.002030 | 0.001124 | 0.002890 | 0.199813 | 0.091299 |
| 0.5 < Q ≤ 0.3 | 0.066850 | 0.052870 | 0.001973 | 0.001043 | 0.000856 | 0.000768 |
| 0.3 < Q ≤ 0.2 | 0.017113 | 0.011815 | 0.010873 | 0.010551 | 0.084693 | 0.036953 |
| Q ≤ 0.1 | 0.026744 | 0.021008 | 0.034137 | 0.020317 | 0.015746 | 0.012960 |

**Tab. 23:** TDE deviation in CTM model at different RTML evaluated by d_L2_ at 25° C

| TDE deviation – CTM model - D_MAX_ | | | | | | |
| --- | --- | --- | --- | --- | --- | --- |
| TA = 25° C, R = 0.5 cm | | | | | | |
|  | SP5 | SP3 | SP1 | SP6 | SP4 | SP2 |
| 0.5 ≤ Q < 1 | 0.096924 | 0.087224 | 0.064009 | 0.047629 | 0.478770 | 0.472093 |
| 0.5 < Q ≤ 0.3 | 0.433839 | 0.420362 | 0.044949 | 0.043581 | 0.038244 | 0.035446 |
| 0.3 < Q ≤ 0.2 | 0.155922 | 0.146795 | 0.119631 | 0.102337 | 0.336467 | 0.331520 |
| Q ≤ 0.1 | 0.305825 | 0.297609 | 0.163489 | 0.160196 | 0.144541 | 0.137588 |
| TA = 25° C, R = 1 cm | | | | | | |
| 0.5 ≤ Q < 1 | 0.200676 | 0.176616 | 0.127789 | 0.093306 | 1.085921 | 1.064973 |
| 0.5 < Q ≤ 0.3 | 0.978343 | 0.949218 | 0.070626 | 0.067887 | 0.059601 | 0.055385 |
| 0.3 < Q ≤ 0.2 | 0.348042 | 0.325264 | 0.267879 | 0.232388 | 0.657130 | 0.643892 |
| Q ≤ 0.1 | 0.594666 | 0.580189 | 0.347716 | 0.338657 | 0.305722 | 0.291640 |
| TA = 25° C, R = 2 cm | | | | | | |
| 0.5 ≤ Q < 1 | 0.401715 | 0.336522 | 0.240250 | 0.171164 | 2.953641 | 2.854130 |
| 0.5 < Q ≤ 0.3 | 2.631010 | 2.552613 | 0.054170 | 0.050111 | 0.042954 | 0.038911 |
| 0.3 < Q ≤ 0.2 | 0.813108 | 0.743778 | 0.622384 | 0.548695 | 1.153148 | 1.114621 |
| Q ≤ 0.1 | 1.035954 | 1.013757 | 0.765055 | 0.732501 | 0.663553 | 0.634064 |

**Tab. 24:** TDE deviation in CTM model at different RTML evaluated by D_MAX_ at 25° C

| TDE deviation – CTM model - d_MAX_ | | | | | | |
| --- | --- | --- | --- | --- | --- | --- |
| TA = 25° C, R = 0.5 cm | | | | | | |
|  | SP5 | SP3 | SP1 | SP6 | SP4 | SP2 |
| 0.5 ≤ Q < 1 | 0.001212 | 0.001182 | 0.001011 | 0.000867 | 0.006383 | 0.006834 |
| 0.5 < Q ≤ 0.3 | 0.007628 | 0.009942 | 0.000591 | 0.000604 | 0.000645 | 0.000759 |
| 0.3 < Q ≤ 0.2 | 0.001994 | 0.001988 | 0.001887 | 0.001859 | 0.004498 | 0.004836 |
| Q ≤ 0.1 | 0.005424 | 0.007133 | 0.002168 | 0.002279 | 0.002499 | 0.003123 |
| TA = 25° C, R = 1 cm | | | | | | |
| 0.5 ≤ Q < 1 | 0.002507 | 0.002418 | 0.002043 | 0.001724 | 0.014545 | 0.015594 |
| 0.5 < Q ≤ 0.3 | 0.017460 | 0.022840 | 0.000930 | 0.000953 | 0.001022 | 0.001211 |
| 0.3 < Q ≤ 0.2 | 0.004466 | 0.004450 | 0.004279 | 0.004279 | 0.008833 | 0.009527 |
| Q ≤ 0.1 | 0.010734 | 0.014195 | 0.004627 | 0.004874 | 0.005365 | 0.006727 |
| TA = 25° C, R = 2 cm | | | | | | |
| 0.5 ≤ Q < 1 | 0.005021 | 0.004729 | 0.003965 | 0.003287 | 0.040067 | 0.043348 |
| 0.5 < Q ≤ 0.3 | 0.048831 | 0.064628 | 0.000704 | 0.000708 | 0.000744 | 0.000843 |
| 0.3 < Q ≤ 0.2 | 0.010484 | 0.010427 | 0.010440 | 0.010801 | 0.015677 | 0.017086 |
| Q ≤ 0.1 | 0.019417 | 0.026088 | 0.010256 | 0.010886 | 0.012065 | 0.015240 |

**Tab. 25:** TDE deviation in CTM model at different RTML evaluated by d_MAX_ at 25° C

| TDE deviation – CMS model - D_L2_ | | | | | | |
| --- | --- | --- | --- | --- | --- | --- |
| TA = 5° C, R = 0.5 cm | | | | | | |
|  | SP5 | SP3 | SP1 | SP6 | SP4 | SP2 |
| 0.5 ≤ Q < 1 | 0.032640 | 0.063827 | 0.088176 | 0.129134 | 0.157386 | 0.121526 |
| 0.5 < Q ≤ 0.3 | 0.088471 | 0.038300 | 0.013530 | 0.020909 | 0.022948 | 0.025622 |
| 0.3 < Q ≤ 0.2 | 0.050525 | 0.086033 | 0.113189 | 0.158891 | 0.022153 | 0.007892 |
| Q ≤ 0.1 | 0.030875 | 0.097214 | 0.013530 | 0.020909 | 0.022948 | 0.025622 |
| TA = 5° C, R = 1 cm | | | | | | |
| 0.5 ≤ Q < 1 | 0.066305 | 0.128104 | 0.177139 | 0.258929 | 0.315048 | 0.245272 |
| 0.5 < Q ≤ 0.3 | 0.177836 | 0.076623 | 0.027342 | 0.041869 | 0.046013 | 0.051357 |
| 0.3 < Q ≤ 0.2 | 0.101746 | 0.172641 | 0.227069 | 0.319241 | 0.044529 | 0.015805 |
| Q ≤ 0.1 | 0.062175 | 0.194561 | 0.027342 | 0.041869 | 0.046013 | 0.051357 |
| TA = 5° C, R = 2 cm | | | | | | |
| 0.5 ≤ Q < 1 | 0.137369 | 0.259809 | 0.357575 | 0.522061 | 0.685682 | 0.544207 |
| 0.5 < Q ≤ 0.3 | 0.403217 | 0.178082 | 0.067248 | 0.100348 | 0.109907 | 0.122891 |
| 0.3 < Q ≤ 0.2 | 0.207394 | 0.348707 | 0.458949 | 0.646399 | 0.043294 | 0.058307 |
| Q ≤ 0.1 | 0.167906 | 0.432219 | 0.067248 | 0.100348 | 0.109907 | 0.122891 |

**Tab. 26:** TDE deviation in CMS model at different RTML evaluated by D_L2_ at 5° C

| TDE deviation – CMS model - d_L2_ | | | | | | |
| --- | --- | --- | --- | --- | --- | --- |
| TA = 5° C, R = 0.5 cm | | | | | | |
|  | SP5 | SP3 | SP1 | SP6 | SP4 | SP2 |
| 0.5 ≤ Q < 1 | 0.003146 | 0.002797 | 0.002718 | 0.002774 | 0.024405 | 0.005645 |
| 0.5 < Q ≤ 0.3 | 0.002819 | 0.000934 | 0.001291 | 0.000939 | 0.000716 | 0.000564 |
| 0.3 < Q ≤ 0.2 | 0.005555 | 0.003815 | 0.003515 | 0.003442 | 0.003021 | 0.000355 |
| Q ≤ 0.1 | 0.000924 | 0.002012 | 0.001291 | 0.000939 | 0.000716 | 0.000564 |
| TA = 5° C, R = 1 cm | | | | | | |
| 0.5 ≤ Q < 1 | 0.006287 | 0.005574 | 0.005427 | 0.005531 | 0.045902 | 0.011368 |
| 0.5 < Q ≤ 0.3 | 0.005650 | 0.001854 | 0.002584 | 0.001872 | 0.001432 | 0.001127 |
| 0.3 < Q ≤ 0.2 | 0.010886 | 0.007644 | 0.007049 | 0.006917 | 0.006050 | 0.000702 |
| Q ≤ 0.1 | 0.001856 | 0.004026 | 0.002584 | 0.001872 | 0.001432 | 0.001127 |
| TA = 5° C, R = 2 cm | | | | | | |
| 0.5 ≤ Q < 1 | 0.012514 | 0.011110 | 0.010795 | 0.010999 | 0.089844 | 0.024893 |
| 0.5 < Q ≤ 0.3 | 0.012638 | 0.004291 | 0.006238 | 0.004440 | 0.003393 | 0.002677 |
| 0.3 < Q ≤ 0.2 | 0.020935 | 0.015330 | 0.014190 | 0.013966 | 0.005571 | 0.002290 |
| Q ≤ 0.1 | 0.005038 | 0.008977 | 0.006238 | 0.004440 | 0.003393 | 0.002677 |

**Tab. 27:** TDE deviation in CMS model at different RTML evaluated by d_L2_ at 5° C

| TDE deviation – CMS model - D_MAX_ | | | | | | |
| --- | --- | --- | --- | --- | --- | --- |
| TA = 5° C, R = 0.5 cm | | | | | | |
|  | SP5 | SP3 | SP1 | SP6 | SP4 | SP2 |
| 0.5 ≤ Q < 1 | 0.278668 | 0.428958 | 0.036924 | 0.335980 | 0.499407 | 0.036924 |
| 0.5 < Q ≤ 0.3 | 0.237840 | 0.249328 | 0.034020 | 0.287842 | 0.348655 | 0.034020 |
| 0.3 < Q ≤ 0.2 | 0.187893 | 0.116238 | 0.029856 | 0.228624 | 0.222521 | 0.029856 |
| Q ≤ 0.1 | 0.154688 | 0.074618 | 0.027514 | 0.190183 | 0.145349 | 0.027514 |
| TA = 5° C, R = 1 cm | | | | | | |
| 0.5 ≤ Q < 1 | 0.554121 | 0.847941 | 0.073925 | 0.676501 | 1.002985 | 0.073925 |
| 0.5 < Q ≤ 0.3 | 0.459769 | 0.466711 | 0.066970 | 0.564490 | 0.663992 | 0.066970 |
| 0.3 < Q ≤ 0.2 | 0.363333 | 0.208421 | 0.059023 | 0.449124 | 0.421534 | 0.059023 |
| Q ≤ 0.1 | 0.298674 | 0.161567 | 0.054472 | 0.373160 | 0.273236 | 0.054472 |
| TA = 5° C, R = 2 cm | | | | | | |
| 0.5 ≤ Q < 1 | 1.095381 | 1.563284 | 0.176399 | 1.371045 | 1.999290 | 0.176399 |
| 0.5 < Q ≤ 0.3 | 0.860177 | 0.756267 | 0.154756 | 1.086690 | 1.247146 | 0.154756 |
| 0.3 < Q ≤ 0.2 | 0.682823 | 0.273074 | 0.137774 | 0.868890 | 0.800136 | 0.137774 |
| Q ≤ 0.1 | 0.557965 | 0.424460 | 0.127253 | 0.719497 | 0.524725 | 0.127253 |

**Tab. 28:** TDE deviation in CMS model at different RTML evaluated by D_MAX_ at 5° C

| TDE deviation – CMS model - d_MAX_ | | | | | | |
| --- | --- | --- | --- | --- | --- | --- |
| TA = 5° C, R = 0.5 cm | | | | | | |
|  | SP5 | SP3 | SP1 | SP6 | SP4 | SP2 |
| 0.5 ≤ Q < 1 | 0.003373 | 0.004851 | 0.000462 | 0.004067 | 0.005769 | 0.000462 |
| 0.5 < Q ≤ 0.3 | 0.003237 | 0.003386 | 0.000472 | 0.003911 | 0.004742 | 0.000472 |
| 0.3 < Q ≤ 0.2 | 0.002975 | 0.001837 | 0.000506 | 0.003612 | 0.003519 | 0.000506 |
| Q ≤ 0.1 | 0.002854 | 0.002088 | 0.000659 | 0.003495 | 0.002675 | 0.000659 |
| TA = 5° C, R = 1 cm | | | | | | |
| 0.5 ≤ Q < 1 | 0.006707 | 0.009717 | 0.000926 | 0.008190 | 0.011565 | 0.000926 |
| 0.5 < Q ≤ 0.3 | 0.006365 | 0.006439 | 0.000951 | 0.007792 | 0.009182 | 0.000951 |
| 0.3 < Q ≤ 0.2 | 0.005875 | 0.003358 | 0.001026 | 0.007224 | 0.006795 | 0.001026 |
| Q ≤ 0.1 | 0.005651 | 0.004713 | 0.001348 | 0.007004 | 0.005139 | 0.001348 |
| TA = 5° C, R = 2 cm | | | | | | |
| 0.5 ≤ Q < 1 | 0.013262 | 0.018462 | 0.002210 | 0.016602 | 0.023619 | 0.002210 |
| 0.5 < Q ≤ 0.3 | 0.012321 | 0.010772 | 0.002306 | 0.015475 | 0.017810 | 0.002306 |
| 0.3 < Q ≤ 0.2 | 0.011503 | 0.004576 | 0.002506 | 0.014483 | 0.013389 | 0.002506 |
| Q ≤ 0.1 | 0.011095 | 0.013574 | 0.003388 | 0.014074 | 0.010307 | 0.003388 |

**Tab. 29:** TDE deviation in CMS model at different RTML evaluated by d_MAX_ at 5° C

| TDE deviation – CMS model - D_L2_ | | | | | | |
| --- | --- | --- | --- | --- | --- | --- |
| TA = 15° C, R = 0.5 cm | | | | | | |
|  | SP5 | SP3 | SP1 | SP6 | SP4 | SP2 |
| 0.5 ≤ Q < 1 | 0.034161 | 0.065649 | 0.090688 | 0.132654 | 0.153244 | 0.120193 |
| 0.5 < Q ≤ 0.3 | 0.088256 | 0.038362 | 0.013462 | 0.021061 | 0.023424 | 0.026482 |
| 0.3 < Q ≤ 0.2 | 0.052706 | 0.088434 | 0.116095 | 0.163142 | 0.018597 | 0.008713 |
| Q ≤ 0.1 | 0.034057 | 0.100654 | 0.013462 | 0.021061 | 0.023424 | 0.026482 |
| TA = 15° C, R = 1 cm | | | | | | |
| 0.5 ≤ Q < 1 | 0.069285 | 0.132029 | 0.182128 | 0.265958 | 0.306829 | 0.242514 |
| 0.5 < Q ≤ 0.3 | 0.177172 | 0.076771 | 0.027210 | 0.042171 | 0.046970 | 0.053053 |
| 0.3 < Q ≤ 0.2 | 0.106043 | 0.177445 | 0.233246 | 0.327834 | 0.037393 | 0.017604 |
| Q ≤ 0.1 | 0.068533 | 0.201491 | 0.027210 | 0.042171 | 0.046970 | 0.053053 |
| TA = 15° C, R = 2 cm | | | | | | |
| 0.5 ≤ Q < 1 | 0.143460 | 0.267320 | 0.367455 | 0.535880 | 0.670457 | 0.539425 |
| 0.5 < Q ≤ 0.3 | 0.403376 | 0.180699 | 0.066939 | 0.100984 | 0.112363 | 0.126849 |
| 0.3 < Q ≤ 0.2 | 0.215966 | 0.358047 | 0.471632 | 0.664137 | 0.030304 | 0.070055 |
| Q ≤ 0.1 | 0.182002 | 0.447997 | 0.066939 | 0.100984 | 0.112363 | 0.126849 |

**Tab. 30:** TDE deviation in CMS model at different RTML evaluated by D_L2_ at 15° C

| TDE deviation – CMS model - d_L2_ | | | | | | |
| --- | --- | --- | --- | --- | --- | --- |
| TA = 15° C, R = 0.5 cm | | | | | | |
|  | SP5 | SP3 | SP1 | SP6 | SP4 | SP2 |
| 0.5 ≤ Q < 1 | 0.003375 | 0.002897 | 0.002806 | 0.002849 | 0.023756 | 0.005618 |
| 0.5 < Q ≤ 0.3 | 0.002822 | 0.000938 | 0.001295 | 0.000951 | 0.000734 | 0.000582 |
| 0.3 < Q ≤ 0.2 | 0.005988 | 0.003949 | 0.003619 | 0.003533 | 0.002517 | 0.000358 |
| Q ≤ 0.1 | 0.001026 | 0.002087 | 0.001295 | 0.000951 | 0.000734 | 0.000582 |
| TA = 15° C, R = 1 cm | | | | | | |
| 0.5 ≤ Q < 1 | 0.006730 | 0.005785 | 0.005601 | 0.005679 | 0.044826 | 0.011312 |
| 0.5 < Q ≤ 0.3 | 0.005649 | 0.001864 | 0.002593 | 0.001897 | 0.001467 | 0.001163 |
| 0.3 < Q ≤ 0.2 | 0.011700 | 0.007913 | 0.007269 | 0.007100 | 0.005058 | 0.000717 |
| Q ≤ 0.1 | 0.002061 | 0.004175 | 0.002593 | 0.001897 | 0.001467 | 0.001163 |
| TA = 15° C, R = 2 cm | | | | | | |
| 0.5 ≤ Q < 1 | 0.013365 | 0.011508 | 0.011134 | 0.011287 | 0.088382 | 0.024838 |
| 0.5 < Q ≤ 0.3 | 0.012691 | 0.004363 | 0.006261 | 0.004494 | 0.003481 | 0.002760 |
| 0.3 < Q ≤ 0.2 | 0.022384 | 0.015854 | 0.014639 | 0.014345 | 0.003881 | 0.002813 |
| Q ≤ 0.1 | 0.005494 | 0.009317 | 0.006261 | 0.004494 | 0.003481 | 0.002760 |

**Tab. 31:** TDE deviation in CMS model at different RTML evaluated by d_L2_ at 15° C

| TDE deviation – CMS model - D_MAX_ | | | | | | |
| --- | --- | --- | --- | --- | --- | --- |
| TA = 15° C, R = 0.5 cm | | | | | | |
|  | SP5 | SP3 | SP1 | SP6 | SP4 | SP2 |
| 0.5 ≤ Q < 1 | 0.267230 | 0.357906 | 0.037319 | 0.322041 | 0.439618 | 0.037319 |
| 0.5 < Q ≤ 0.3 | 0.247672 | 0.240927 | 0.036283 | 0.299441 | 0.353170 | 0.036283 |
| 0.3 < Q ≤ 0.2 | 0.196644 | 0.114936 | 0.031731 | 0.239472 | 0.230824 | 0.031731 |
| Q ≤ 0.1 | 0.158513 | 0.073921 | 0.028449 | 0.194800 | 0.149584 | 0.028449 |
| TA = 15° C, R = 1 cm | | | | | | |
| 0.5 ≤ Q < 1 | 0.531431 | 0.717470 | 0.074719 | 0.648396 | 0.881242 | 0.074719 |
| 0.5 < Q ≤ 0.3 | 0.482692 | 0.458454 | 0.071888 | 0.592171 | 0.682468 | 0.071888 |
| 0.3 < Q ≤ 0.2 | 0.380752 | 0.209685 | 0.062621 | 0.471052 | 0.440656 | 0.062621 |
| Q ≤ 0.1 | 0.307615 | 0.158411 | 0.056403 | 0.384086 | 0.284623 | 0.056403 |
| TA = 15° C, R = 2 cm | | | | | | |
| 0.5 ≤ Q < 1 | 1.050738 | 1.369435 | 0.178258 | 1.313951 | 1.804971 | 0.178258 |
| 0.5 < Q ≤ 0.3 | 0.915332 | 0.762669 | 0.167574 | 1.155611 | 1.314195 | 0.167574 |
| 0.3 < Q ≤ 0.2 | 0.715042 | 0.285432 | 0.145382 | 0.911878 | 0.848165 | 0.145382 |
| Q ≤ 0.1 | 0.579908 | 0.414947 | 0.132116 | 0.746680 | 0.559106 | 0.132116 |

**Tab. 32:** TDE deviation in CMS model at different RTML evaluated by D_MAX_ at 15° C

| TDE deviation – CMS model - d_MAX_ | | | | | | |
| --- | --- | --- | --- | --- | --- | --- |
| TA = 15° C, R = 0.5 cm | | | | | | |
|  | SP5 | SP3 | SP1 | SP6 | SP4 | SP2 |
| 0.5 ≤ Q < 1 | 0.003380 | 0.004145 | 0.000491 | 0.004076 | 0.005274 | 0.000491 |
| 0.5 < Q ≤ 0.3 | 0.003354 | 0.003258 | 0.000499 | 0.004050 | 0.004782 | 0.000499 |
| 0.3 < Q ≤ 0.2 | 0.003098 | 0.001807 | 0.000521 | 0.003763 | 0.003631 | 0.000521 |
| Q ≤ 0.1 | 0.002910 | 0.002067 | 0.000672 | 0.003563 | 0.002738 | 0.000672 |
| TA = 15° C, R = 1 cm | | | | | | |
| 0.5 ≤ Q < 1 | 0.006723 | 0.008310 | 0.000986 | 0.008207 | 0.010572 | 0.000986 |
| 0.5 < Q ≤ 0.3 | 0.006635 | 0.006281 | 0.001001 | 0.008116 | 0.009368 | 0.001001 |
| 0.3 < Q ≤ 0.2 | 0.006105 | 0.003335 | 0.001052 | 0.007513 | 0.007043 | 0.001052 |
| Q ≤ 0.1 | 0.005767 | 0.004590 | 0.001369 | 0.007144 | 0.005306 | 0.001369 |
| TA = 15° C, R = 2 cm | | | | | | |
| 0.5 ≤ Q < 1 | 0.013299 | 0.016029 | 0.002362 | 0.016633 | 0.021709 | 0.002362 |
| 0.5 < Q ≤ 0.3 | 0.012952 | 0.010729 | 0.002399 | 0.016258 | 0.018541 | 0.002399 |
| 0.3 < Q ≤ 0.2 | 0.011877 | 0.004713 | 0.002561 | 0.014988 | 0.013990 | 0.002561 |
| Q ≤ 0.1 | 0.011353 | 0.013030 | 0.003420 | 0.014420 | 0.010808 | 0.003420 |

**Tab. 33:** TDE deviation in CMS model at different RTML evaluated by d_MAX_ at 15° C

| TDE deviation – CMS model - D_L2_ | | | | | | |
| --- | --- | --- | --- | --- | --- | --- |
| TA = 25° C, R = 0.5 cm | | | | | | |
|  | SP5 | SP3 | SP1 | SP6 | SP4 | SP2 |
| 0.5 ≤ Q < 1 | 0.038478 | 0.071490 | 0.098038 | 0.142283 | 0.148087 | 0.123710 |
| 0.5 < Q ≤ 0.3 | 0.094223 | 0.043286 | 0.013333 | 0.021463 | 0.024408 | 0.028127 |
| 0.3 < Q ≤ 0.2 | 0.059005 | 0.095716 | 0.124712 | 0.174265 | 0.014738 | 0.009184 |
| Q ≤ 0.1 | 0.034987 | 0.101611 | 0.013333 | 0.021463 | 0.024408 | 0.028127 |
| TA = 25° C, R = 1 cm | | | | | | |
| 0.5 ≤ Q < 1 | 0.077993 | 0.143745 | 0.196397 | 0.285325 | 0.296965 | 0.249710 |
| 0.5 < Q ≤ 0.3 | 0.189670 | 0.086183 | 0.026925 | 0.042994 | 0.048910 | 0.056394 |
| 0.3 < Q ≤ 0.2 | 0.118622 | 0.191929 | 0.250419 | 0.350618 | 0.029610 | 0.018628 |
| Q ≤ 0.1 | 0.070344 | 0.203466 | 0.026925 | 0.042994 | 0.048910 | 0.056394 |
| TA = 25° C, R = 2 cm | | | | | | |
| 0.5 ≤ Q < 1 | 0.160933 | 0.291063 | 0.395660 | 0.575337 | 0.652367 | 0.556694 |
| 0.5 < Q ≤ 0.3 | 0.432286 | 0.207759 | 0.066232 | 0.102929 | 0.117140 | 0.134956 |
| 0.3 < Q ≤ 0.2 | 0.241112 | 0.387079 | 0.506973 | 0.710300 | 0.018164 | 0.076201 |
| Q ≤ 0.1 | 0.188146 | 0.456064 | 0.066232 | 0.102929 | 0.117140 | 0.134956 |

**Tab. 34:** TDE deviation in CMS model at different RTML evaluated by D_L2_ at 25° C

| TDE deviation – CMS model - d_L2_ | | | | | | |
| --- | --- | --- | --- | --- | --- | --- |
| TA = 25° C, R = 0.5 cm | | | | | | |
|  | SP5 | SP3 | SP1 | SP6 | SP4 | SP2 |
| 0.5 ≤ Q < 1 | 0.003969 | 0.003170 | 0.003020 | 0.003014 | 0.022663 | 0.005798 |
| 0.5 < Q ≤ 0.3 | 0.002996 | 0.001057 | 0.001297 | 0.000971 | 0.000761 | 0.000609 |
| 0.3 < Q ≤ 0.2 | 0.007127 | 0.004297 | 0.003872 | 0.003725 | 0.001879 | 0.000368 |
| Q ≤ 0.1 | 0.001050 | 0.002078 | 0.001297 | 0.000971 | 0.000761 | 0.000609 |
| TA = 25° C, R = 1 cm | | | | | | |
| 0.5 ≤ Q < 1 | 0.007905 | 0.006328 | 0.006013 | 0.006009 | 0.043089 | 0.011684 |
| 0.5 < Q ≤ 0.3 | 0.006015 | 0.002096 | 0.002595 | 0.001938 | 0.001520 | 0.001218 |
| 0.3 < Q ≤ 0.2 | 0.013868 | 0.008605 | 0.007773 | 0.007495 | 0.003778 | 0.000741 |
| Q ≤ 0.1 | 0.002108 | 0.004158 | 0.002595 | 0.001938 | 0.001520 | 0.001218 |
| TA = 25° C, R = 2 cm | | | | | | |
| 0.5 ≤ Q < 1 | 0.015622 | 0.012586 | 0.011935 | 0.011947 | 0.086263 | 0.025747 |
| 0.5 < Q ≤ 0.3 | 0.013542 | 0.004975 | 0.006268 | 0.004592 | 0.003611 | 0.002894 |
| 0.3 < Q ≤ 0.2 | 0.026317 | 0.017237 | 0.015677 | 0.015145 | 0.002493 | 0.003095 |
| Q ≤ 0.1 | 0.005657 | 0.009355 | 0.006268 | 0.004592 | 0.003611 | 0.002894 |

**Tab. 35:** TDE deviation in CMS model at different RTML evaluated by d_L2_ at 25° C

| TDE deviation – CMS model - D_MAX_ | | | | | | |
| --- | --- | --- | --- | --- | --- | --- |
| TA = 25° C, R = 0.5 cm | | | | | | |
|  | SP5 | SP3 | SP1 | SP6 | SP4 | SP2 |
| 0.5 ≤ Q < 1 | 0.262335 | 0.305714 | 0.037699 | 0.315216 | 0.395636 | 0.037699 |
| 0.5 < Q ≤ 0.3 | 0.246257 | 0.194035 | 0.036956 | 0.296862 | 0.319504 | 0.036956 |
| 0.3 < Q ≤ 0.2 | 0.201215 | 0.085054 | 0.033200 | 0.244457 | 0.214002 | 0.033200 |
| Q ≤ 0.1 | 0.168303 | 0.085014 | 0.030656 | 0.206545 | 0.144324 | 0.030656 |
| TA = 25° C, R = 1 cm | | | | | | |
| 0.5 ≤ Q < 1 | 0.521653 | 0.610921 | 0.075482 | 0.634763 | 0.792969 | 0.075482 |
| 0.5 < Q ≤ 0.3 | 0.482290 | 0.371160 | 0.073451 | 0.589914 | 0.621087 | 0.073451 |
| 0.3 < Q ≤ 0.2 | 0.393083 | 0.155605 | 0.065927 | 0.484524 | 0.413476 | 0.065927 |
| Q ≤ 0.1 | 0.327041 | 0.179284 | 0.060712 | 0.408028 | 0.276591 | 0.060712 |
| TA = 25° C, R = 2 cm | | | | | | |
| 0.5 ≤ Q < 1 | 1.031287 | 1.148291 | 0.180043 | 1.286870 | 1.631598 | 0.180043 |
| 0.5 < Q ≤ 0.3 | 0.923440 | 0.610350 | 0.172432 | 1.163235 | 1.221785 | 0.172432 |
| 0.3 < Q ≤ 0.2 | 0.751407 | 0.193555 | 0.154966 | 0.953344 | 0.821985 | 0.154966 |
| Q ≤ 0.1 | 0.617182 | 0.448666 | 0.141694 | 0.796183 | 0.555555 | 0.141694 |

**Tab. 36:** TDE deviation in CMS model at different RTML evaluated by D_MAX_ at 25° C

| TDE deviation – CMS model - d_MAX_ | | | | | | |
| --- | --- | --- | --- | --- | --- | --- |
| TA = 25° C, R = 0.5 cm | | | | | | |
|  | SP5 | SP3 | SP1 | SP6 | SP4 | SP2 |
| 0.5 ≤ Q < 1 | 0.003334 | 0.003476 | 0.000498 | 0.004011 | 0.004720 | 0.000498 |
| 0.5 < Q ≤ 0.3 | 0.003327 | 0.002617 | 0.000521 | 0.004005 | 0.004315 | 0.000521 |
| 0.3 < Q ≤ 0.2 | 0.003169 | 0.001336 | 0.000563 | 0.003838 | 0.003365 | 0.000563 |
| Q ≤ 0.1 | 0.003101 | 0.002432 | 0.000715 | 0.003798 | 0.002652 | 0.000715 |
| TA = 25° C, R = 1 cm | | | | | | |
| 0.5 ≤ Q < 1 | 0.006631 | 0.006973 | 0.001003 | 0.008077 | 0.009461 | 0.001003 |
| 0.5 < Q ≤ 0.3 | 0.006599 | 0.005062 | 0.001051 | 0.008049 | 0.008490 | 0.001051 |
| 0.3 < Q ≤ 0.2 | 0.006286 | 0.002479 | 0.001135 | 0.007707 | 0.006592 | 0.001135 |
| Q ≤ 0.1 | 0.006142 | 0.005296 | 0.001458 | 0.007657 | 0.005164 | 0.001458 |
| TA = 25° C, R = 2 cm | | | | | | |
| 0.5 ≤ Q < 1 | 0.013115 | 0.013349 | 0.002410 | 0.016376 | 0.019532 | 0.002410 |
| 0.5 < Q ≤ 0.3 | 0.012962 | 0.008513 | 0.002539 | 0.016224 | 0.017095 | 0.002539 |
| 0.3 < Q ≤ 0.2 | 0.012393 | 0.003171 | 0.002736 | 0.015559 | 0.013462 | 0.002736 |
| Q ≤ 0.1 | 0.012044 | 0.014247 | 0.003610 | 0.015574 | 0.010705 | 0.003610 |

**Tab. 37:** TDE deviation in CMS model at different RTML evaluated by d_MAX_ at 25° C

| TDE deviation – CM model - D_L2_ | | | | | | |
| --- | --- | --- | --- | --- | --- | --- |
| TA = 5° C, R = 0.5 cm | | | | | | |
|  | SP5 | SP3 | SP1 | SP6 | SP4 | SP2 |
| 0.5 ≤ Q < 1 | 0.043480 | 0.080502 | 0.102427 | 0.126927 | 0.083246 | 0.071477 |
| 0.5 < Q ≤ 0.3 | 0.070044 | 0.070940 | 0.013787 | 0.020148 | 0.021296 | 0.022356 |
| 0.3 < Q ≤ 0.2 | 0.066211 | 0.106579 | 0.129449 | 0.155221 | 0.044187 | 0.044887 |
| Q ≤ 0.1 | 0.045741 | 0.047726 | 0.013787 | 0.020148 | 0.021296 | 0.022356 |
| TA = 5° C, R = 1 cm | | | | | | |
| 0.5 ≤ Q < 1 | 0.087742 | 0.161368 | 0.204658 | 0.253615 | 0.165979 | 0.143371 |
| 0.5 < Q ≤ 0.3 | 0.140651 | 0.142356 | 0.027705 | 0.040295 | 0.042655 | 0.044755 |
| 0.3 < Q ≤ 0.2 | 0.132465 | 0.213645 | 0.259416 | 0.311667 | 0.088234 | 0.089981 |
| Q ≤ 0.1 | 0.091728 | 0.095658 | 0.027705 | 0.040295 | 0.042655 | 0.044755 |
| TA = 5° C, R = 2 cm | | | | | | |
| 0.5 ≤ Q < 1 | 0.179378 | 0.324402 | 0.409727 | 0.505388 | 0.382934 | 0.337594 |
| 0.5 < Q ≤ 0.3 | 0.332311 | 0.336673 | 0.067297 | 0.096366 | 0.101724 | 0.106779 |
| 0.3 < Q ≤ 0.2 | 0.266240 | 0.428593 | 0.522430 | 0.629113 | 0.222108 | 0.222312 |
| Q ≤ 0.1 | 0.225004 | 0.233879 | 0.067297 | 0.096366 | 0.101724 | 0.106779 |

**Tab. 38:** TDE deviation in CM model at different RTML evaluated by D_L2_ at 5° C

| TDE deviation – CM model - d_L2_ | | | | | | |
| --- | --- | --- | --- | --- | --- | --- |
| TA = 5° C, R = 0.5 cm | | | | | | |
|  | SP5 | SP3 | SP1 | SP6 | SP4 | SP2 |
| 0.5 ≤ Q < 1 | 0.003681 | 0.003077 | 0.002829 | 0.002618 | 0.012821 | 0.002820 |
| 0.5 < Q ≤ 0.3 | 0.001957 | 0.001485 | 0.001172 | 0.000786 | 0.000594 | 0.000467 |
| 0.3 < Q ≤ 0.2 | 0.006740 | 0.004121 | 0.003602 | 0.003225 | 0.006419 | 0.001762 |
| Q ≤ 0.1 | 0.001276 | 0.000997 | 0.001172 | 0.000786 | 0.000594 | 0.000467 |
| TA = 5° C, R = 1 cm | | | | | | |
| 0.5 ≤ Q < 1 | 0.007359 | 0.006141 | 0.005631 | 0.005214 | 0.024418 | 0.005657 |
| 0.5 < Q ≤ 0.3 | 0.003931 | 0.002980 | 0.002344 | 0.001571 | 0.001188 | 0.000934 |
| 0.3 < Q ≤ 0.2 | 0.013188 | 0.008277 | 0.007234 | 0.006489 | 0.012287 | 0.003531 |
| Q ≤ 0.1 | 0.002559 | 0.001998 | 0.002344 | 0.001571 | 0.001188 | 0.000934 |
| TA = 5° C, R = 2 cm | | | | | | |
| 0.5 ≤ Q < 1 | 0.014687 | 0.012221 | 0.011177 | 0.010315 | 0.051518 | 0.013297 |
| 0.5 < Q ≤ 0.3 | 0.009276 | 0.007041 | 0.005666 | 0.003746 | 0.002829 | 0.002226 |
| 0.3 < Q ≤ 0.2 | 0.025253 | 0.016636 | 0.014610 | 0.013138 | 0.028731 | 0.008712 |
| Q ≤ 0.1 | 0.006269 | 0.004882 | 0.005666 | 0.003746 | 0.002829 | 0.002226 |

**Tab. 39:** TDE deviation in CM model at different RTML evaluated by d_L2_ at 5° C

| TDE deviation – CM model - D_MAX_ | | | | | | |
| --- | --- | --- | --- | --- | --- | --- |
| TA = 5° C, R = 0.5 cm | | | | | | |
|  | SP5 | SP3 | SP1 | SP6 | SP4 | SP2 |
| 0.5 ≤ Q < 1 | 0.200747 | 0.082531 | 0.027118 | 0.234452 | 0.058478 | 0.027118 |
| 0.5 < Q ≤ 0.3 | 0.167312 | 0.075431 | 0.024499 | 0.198540 | 0.052605 | 0.024499 |
| 0.3 < Q ≤ 0.2 | 0.147537 | 0.072658 | 0.023330 | 0.177736 | 0.049917 | 0.023308 |
| Q ≤ 0.1 | 0.132639 | 0.071803 | 0.022760 | 0.162265 | 0.048585 | 0.022760 |
| TA = 5° C, R = 1 cm | | | | | | |
| 0.5 ≤ Q < 1 | 0.398077 | 0.165600 | 0.054291 | 0.471954 | 0.117226 | 0.054291 |
| 0.5 < Q ≤ 0.3 | 0.330775 | 0.151129 | 0.048947 | 0.398347 | 0.105220 | 0.048947 |
| 0.3 < Q ≤ 0.2 | 0.291018 | 0.145433 | 0.046554 | 0.355941 | 0.099732 | 0.046554 |
| Q ≤ 0.1 | 0.261757 | 0.143978 | 0.045487 | 0.324939 | 0.097223 | 0.045487 |
| TA = 5° C, R = 2 cm | | | | | | |
| 0.5 ≤ Q < 1 | 0.781054 | 0.393109 | 0.129297 | 0.954274 | 0.284217 | 0.129297 |
| 0.5 < Q ≤ 0.3 | 0.647372 | 0.357693 | 0.116338 | 0.802568 | 0.255143 | 0.116338 |
| 0.3 < Q ≤ 0.2 | 0.566684 | 0.343067 | 0.110378 | 0.713686 | 0.241735 | 0.110378 |
| Q ≤ 0.1 | 0.509666 | 0.340011 | 0.108049 | 0.651276 | 0.236595 | 0.108049 |

**Tab. 40:** TDE deviation in CM model at different RTML evaluated by D_MAX_ at 5° C

| TDE deviation – CM model - d_MAX_ | | | | | | |
| --- | --- | --- | --- | --- | --- | --- |
| TA = 5° C, R = 0.5 cm | | | | | | |
|  | SP5 | SP3 | SP1 | SP6 | SP4 | SP2 |
| 0.5 ≤ Q < 1 | 0.002352 | 0.001059 | 0.000344 | 0.002785 | 0.000738 | 0.000344 |
| 0.5 < Q ≤ 0.3 | 0.002462 | 0.001209 | 0.000388 | 0.002957 | 0.000831 | 0.000388 |
| 0.3 < Q ≤ 0.2 | 0.002600 | 0.001403 | 0.000445 | 0.003167 | 0.000950 | 0.000445 |
| Q ≤ 0.1 | 0.002857 | 0.001980 | 0.000601 | 0.003625 | 0.001292 | 0.000601 |
| TA = 5° C, R = 1 cm | | | | | | |
| 0.5 ≤ Q < 1 | 0.004688 | 0.002135 | 0.000692 | 0.005623 | 0.001487 | 0.000692 |
| 0.5 < Q ≤ 0.3 | 0.004907 | 0.002441 | 0.000782 | 0.005960 | 0.001674 | 0.000782 |
| 0.3 < Q ≤ 0.2 | 0.005196 | 0.002840 | 0.000898 | 0.006390 | 0.001919 | 0.000898 |
| Q ≤ 0.1 | 0.005730 | 0.004033 | 0.001220 | 0.007316 | 0.002625 | 0.001220 |
| TA = 5° C, R = 2 cm | | | | | | |
| 0.5 ≤ Q < 1 | 0.009320 | 0.005118 | 0.001667 | 0.011462 | 0.003653 | 0.001667 |
| 0.5 < Q ≤ 0.3 | 0.009741 | 0.005845 | 0.001882 | 0.012107 | 0.004120 | 0.001882 |
| 0.3 < Q ≤ 0.2 | 0.010359 | 0.006826 | 0.002173 | 0.012990 | 0.004754 | 0.002173 |
| Q ≤ 0.1 | 0.011468 | 0.009763 | 0.002974 | 0.014851 | 0.006596 | 0.002974 |

**Tab. 41:** TDE deviation in CM model at different RTML evaluated by d_MAX_ at 5° C

| TDE deviation – CM model - D_L2_ | | | | | | |
| --- | --- | --- | --- | --- | --- | --- |
| TA = 15° C, R = 0.5 cm | | | | | | |
|  | SP5 | SP3 | SP1 | SP6 | SP4 | SP2 |
| 0.5 ≤ Q < 1 | 0.044412 | 0.081257 | 0.103109 | 0.127777 | 0.083382 | 0.074205 |
| 0.5 < Q ≤ 0.3 | 0.073472 | 0.075233 | 0.013607 | 0.020184 | 0.021616 | 0.022887 |
| 0.3 < Q ≤ 0.2 | 0.067593 | 0.107447 | 0.130433 | 0.156321 | 0.043686 | 0.044450 |
| Q ≤ 0.1 | 0.045450 | 0.047568 | 0.013607 | 0.020184 | 0.021616 | 0.022887 |
| TA = 15° C, R = 1 cm | | | | | | |
| 0.5 ≤ Q < 1 | 0.089633 | 0.162784 | 0.206202 | 0.255256 | 0.166441 | 0.148788 |
| 0.5 < Q ≤ 0.3 | 0.147692 | 0.150930 | 0.027332 | 0.040437 | 0.043279 | 0.045801 |
| 0.3 < Q ≤ 0.2 | 0.135243 | 0.215089 | 0.261649 | 0.313732 | 0.087286 | 0.089065 |
| Q ≤ 0.1 | 0.091104 | 0.095378 | 0.027332 | 0.040437 | 0.043279 | 0.045801 |
| TA = 15° C, R = 2 cm | | | | | | |
| 0.5 ≤ Q < 1 | 0.183177 | 0.326924 | 0.412510 | 0.508928 | 0.384473 | 0.349492 |
| 0.5 < Q ≤ 0.3 | 0.347543 | 0.355924 | 0.066408 | 0.096640 | 0.103282 | 0.109297 |
| 0.3 < Q ≤ 0.2 | 0.271730 | 0.431779 | 0.525954 | 0.633845 | 0.220291 | 0.221127 |
| Q ≤ 0.1 | 0.225036 | 0.234829 | 0.066408 | 0.096640 | 0.103282 | 0.109297 |

**Tab. 42:** TDE deviation in CM model at different RTML evaluated by D_L2_ at 15° C

| TDE deviation – CM model - d_L2_ | | | | | | |
| --- | --- | --- | --- | --- | --- | --- |
| TA = 15° C, R = 0.5 cm | | | | | | |
|  | SP5 | SP3 | SP1 | SP6 | SP4 | SP2 |
| 0.5 ≤ Q < 1 | 0.003925 | 0.003160 | 0.002890 | 0.002668 | 0.012794 | 0.002976 |
| 0.5 < Q ≤ 0.3 | 0.002083 | 0.001594 | 0.001177 | 0.000800 | 0.000611 | 0.000484 |
| 0.3 < Q ≤ 0.2 | 0.007253 | 0.004228 | 0.003683 | 0.003288 | 0.006489 | 0.001775 |
| Q ≤ 0.1 | 0.001287 | 0.001006 | 0.001177 | 0.000800 | 0.000611 | 0.000484 |
| TA = 15° C, R = 1 cm | | | | | | |
| 0.5 ≤ Q < 1 | 0.007844 | 0.006303 | 0.005757 | 0.005311 | 0.024452 | 0.005970 |
| 0.5 < Q ≤ 0.3 | 0.004188 | 0.003198 | 0.002354 | 0.001602 | 0.001223 | 0.000968 |
| 0.3 < Q ≤ 0.2 | 0.014160 | 0.008480 | 0.007404 | 0.006613 | 0.012445 | 0.003556 |
| Q ≤ 0.1 | 0.002579 | 0.002017 | 0.002354 | 0.001602 | 0.001223 | 0.000968 |
| TA = 15° C, R = 2 cm | | | | | | |
| 0.5 ≤ Q < 1 | 0.015613 | 0.012530 | 0.011416 | 0.010509 | 0.051909 | 0.014003 |
| 0.5 < Q ≤ 0.3 | 0.009847 | 0.007536 | 0.005691 | 0.003818 | 0.002914 | 0.002306 |
| 0.3 < Q ≤ 0.2 | 0.026953 | 0.017055 | 0.014926 | 0.013401 | 0.029119 | 0.008814 |
| Q ≤ 0.1 | 0.006362 | 0.004961 | 0.005691 | 0.003818 | 0.002914 | 0.002306 |

**Tab. 43:** TDE deviation in CM model at different RTML evaluated by d_L2_ at 15° C

| TDE deviation – CM model - D_MAX_ | | | | | | |
| --- | --- | --- | --- | --- | --- | --- |
| TA = 15° C, R = 0.5 cm | | | | | | |
|  | SP5 | SP3 | SP1 | SP6 | SP4 | SP2 |
| 0.5 ≤ Q < 1 | 0.197012 | 0.089669 | 0.028232 | 0.231895 | 0.058956 | 0.028232 |
| 0.5 < Q ≤ 0.3 | 0.175701 | 0.083939 | 0.026275 | 0.208845 | 0.054782 | 0.026275 |
| 0.3 < Q ≤ 0.2 | 0.150650 | 0.077918 | 0.024152 | 0.181378 | 0.050239 | 0.024152 |
| Q ≤ 0.1 | 0.133215 | 0.075424 | 0.023037 | 0.162939 | 0.047882 | 0.023037 |
| TA = 15° C, R = 1 cm | | | | | | |
| 0.5 ≤ Q < 1 | 0.391470 | 0.179959 | 0.056522 | 0.467431 | 0.118182 | 0.056522 |
| 0.5 < Q ≤ 0.3 | 0.346627 | 0.167844 | 0.052371 | 0.418501 | 0.109343 | 0.052371 |
| 0.3 < Q ≤ 0.2 | 0.297808 | 0.156149 | 0.048259 | 0.363749 | 0.100499 | 0.048259 |
| Q ≤ 0.1 | 0.262909 | 0.151386 | 0.045997 | 0.326199 | 0.095721 | 0.045997 |
| TA = 15° C, R = 2 cm | | | | | | |
| 0.5 ≤ Q < 1 | 0.772885 | 0.425441 | 0.134783 | 0.949703 | 0.289311 | 0.134783 |
| 0.5 < Q ≤ 0.3 | 0.675116 | 0.393752 | 0.123877 | 0.840604 | 0.265806 | 0.123877 |
| 0.3 < Q ≤ 0.2 | 0.582480 | 0.367737 | 0.114700 | 0.731941 | 0.245890 | 0.114700 |
| Q ≤ 0.1 | 0.513395 | 0.356835 | 0.109293 | 0.654243 | 0.234786 | 0.109293 |

**Tab. 44:** TDE deviation in CM model at different RTML evaluated by D_MAX_ at 15° C

| TDE deviation – CM model - d_MAX_ | | | | | | |
| --- | --- | --- | --- | --- | --- | --- |
| TA = 15° C, R = 0.5 cm | | | | | | |
|  | SP5 | SP3 | SP1 | SP6 | SP4 | SP2 |
| 0.5 ≤ Q < 1 | 0.002466 | 0.001172 | 0.000367 | 0.002917 | 0.000765 | 0.000367 |
| 0.5 < Q ≤ 0.3 | 0.002493 | 0.001286 | 0.000399 | 0.002992 | 0.000829 | 0.000399 |
| 0.3 < Q ≤ 0.2 | 0.002583 | 0.001454 | 0.000445 | 0.003145 | 0.000926 | 0.000445 |
| Q ≤ 0.1 | 0.002881 | 0.002037 | 0.000600 | 0.003638 | 0.001261 | 0.000600 |
| TA = 15° C, R = 1 cm | | | | | | |
| 0.5 ≤ Q < 1 | 0.004903 | 0.002356 | 0.000736 | 0.005878 | 0.001536 | 0.000736 |
| 0.5 < Q ≤ 0.3 | 0.004973 | 0.002596 | 0.000803 | 0.006036 | 0.001671 | 0.000803 |
| 0.3 < Q ≤ 0.2 | 0.005156 | 0.002935 | 0.000897 | 0.006338 | 0.001866 | 0.000897 |
| Q ≤ 0.1 | 0.005794 | 0.004146 | 0.001218 | 0.007344 | 0.002560 | 0.001218 |
| TA = 15° C, R = 2 cm | | | | | | |
| 0.5 ≤ Q < 1 | 0.009688 | 0.005591 | 0.001761 | 0.011937 | 0.003778 | 0.001761 |
| 0.5 < Q ≤ 0.3 | 0.009903 | 0.006195 | 0.001935 | 0.012282 | 0.004145 | 0.001935 |
| 0.3 < Q ≤ 0.2 | 0.010293 | 0.007015 | 0.002167 | 0.012874 | 0.004648 | 0.002167 |
| Q ≤ 0.1 | 0.011670 | 0.010013 | 0.002976 | 0.014949 | 0.006485 | 0.002976 |

**Tab. 45:** TDE deviation in CM model at different RTML evaluated by d_MAX_ at 15° C

| TDE deviation – CM model - D_L2_ | | | | | | |
| --- | --- | --- | --- | --- | --- | --- |
| TA = 25° C, R = 0.5 cm | | | | | | |
|  | SP5 | SP3 | SP1 | SP6 | SP4 | SP2 |
| 0.5 ≤ Q < 1 | 0.048893 | 0.086247 | 0.109408 | 0.136008 | 0.082495 | 0.078117 |
| 0.5 < Q ≤ 0.3 | 0.079237 | 0.082740 | 0.013421 | 0.020423 | 0.022428 | 0.024277 |
| 0.3 < Q ≤ 0.2 | 0.074043 | 0.113262 | 0.137885 | 0.166062 | 0.043174 | 0.044631 |
| Q ≤ 0.1 | 0.046333 | 0.049296 | 0.013421 | 0.020423 | 0.022428 | 0.024277 |
| TA = 25° C, R = 1 cm | | | | | | |
| 0.5 ≤ Q < 1 | 0.098688 | 0.172683 | 0.218668 | 0.271596 | 0.164905 | 0.156889 |
| 0.5 < Q ≤ 0.3 | 0.158999 | 0.166067 | 0.026971 | 0.040851 | 0.044885 | 0.048638 |
| 0.3 < Q ≤ 0.2 | 0.148096 | 0.227194 | 0.276565 | 0.333474 | 0.086369 | 0.089379 |
| Q ≤ 0.1 | 0.092832 | 0.098803 | 0.026971 | 0.040851 | 0.044885 | 0.048638 |
| TA = 25° C, R = 2 cm | | | | | | |
| 0.5 ≤ Q < 1 | 0.201468 | 0.346813 | 0.438092 | 0.541633 | 0.382464 | 0.367527 |
| 0.5 < Q ≤ 0.3 | 0.373152 | 0.390259 | 0.065528 | 0.097779 | 0.107185 | 0.116083 |
| 0.3 < Q ≤ 0.2 | 0.297398 | 0.456548 | 0.556671 | 0.674115 | 0.218338 | 0.223202 |
| Q ≤ 0.1 | 0.231203 | 0.244985 | 0.065528 | 0.097779 | 0.107185 | 0.116083 |

**Tab. 46:** TDE deviation in CM model at different RTML evaluated by D_L2_ at 25° C

| TDE deviation – CM model - d_L2_ | | | | | | |
| --- | --- | --- | --- | --- | --- | --- |
| TA = 25° C, R = 0.5 cm | | | | | | |
|  | SP5 | SP3 | SP1 | SP6 | SP4 | SP2 |
| 0.5 ≤ Q < 1 | 0.004573 | 0.003400 | 0.003081 | 0.002823 | 0.012065 | 0.003170 |
| 0.5 < Q ≤ 0.3 | 0.002256 | 0.001742 | 0.001182 | 0.000819 | 0.000637 | 0.000510 |
| 0.3 < Q ≤ 0.2 | 0.008418 | 0.004518 | 0.003912 | 0.003473 | 0.006311 | 0.001805 |
| Q ≤ 0.1 | 0.001318 | 0.001036 | 0.001182 | 0.000819 | 0.000637 | 0.000510 |
| TA = 25° C, R = 1 cm | | | | | | |
| 0.5 ≤ Q < 1 | 0.009129 | 0.006775 | 0.006132 | 0.005617 | 0.023237 | 0.006368 |
| 0.5 < Q ≤ 0.3 | 0.004528 | 0.003496 | 0.002363 | 0.001636 | 0.001274 | 0.001021 |
| 0.3 < Q ≤ 0.2 | 0.016398 | 0.009080 | 0.007863 | 0.006989 | 0.012173 | 0.003611 |
| Q ≤ 0.1 | 0.002639 | 0.002076 | 0.002363 | 0.001636 | 0.001274 | 0.001021 |
| TA = 25° C, R = 2 cm | | | | | | |
| 0.5 ≤ Q < 1 | 0.018109 | 0.013454 | 0.012167 | 0.011110 | 0.050206 | 0.014892 |
| 0.5 < Q ≤ 0.3 | 0.010615 | 0.008208 | 0.005707 | 0.003903 | 0.003034 | 0.002432 |
| 0.3 < Q ≤ 0.2 | 0.031091 | 0.018267 | 0.015864 | 0.014165 | 0.028682 | 0.008996 |
| Q ≤ 0.1 | 0.006559 | 0.005140 | 0.005707 | 0.003903 | 0.003034 | 0.002432 |

**Tab. 47:** TDE deviation in CM model at different RTML evaluated by d_L2_ at 25° C

| TDE deviation – CM model - D_MAX_ | | | | | | |
| --- | --- | --- | --- | --- | --- | --- |
| TA = 25° C, R = 0.5 cm | | | | | | |
|  | SP5 | SP3 | SP1 | SP6 | SP4 | SP2 |
| 0.5 ≤ Q < 1 | 0.208154 | 0.103755 | 0.031377 | 0.246592 | 0.063438 | 0.031377 |
| 0.5 < Q ≤ 0.3 | 0.197806 | 0.101076 | 0.030469 | 0.235852 | 0.061586 | 0.030469 |
| 0.3 < Q ≤ 0.2 | 0.163070 | 0.088843 | 0.026560 | 0.196638 | 0.053675 | 0.026560 |
| Q ≤ 0.1 | 0.142644 | 0.083820 | 0.024782 | 0.174261 | 0.050165 | 0.024782 |
| TA = 25° C, R = 1 cm | | | | | | |
| 0.5 ≤ Q < 1 | 0.413721 | 0.208270 | 0.062820 | 0.496892 | 0.127163 | 0.062820 |
| 0.5 < Q ≤ 0.3 | 0.390814 | 0.202239 | 0.060775 | 0.473168 | 0.123023 | 0.060775 |
| 0.3 < Q ≤ 0.2 | 0.321978 | 0.177811 | 0.052972 | 0.394433 | 0.107212 | 0.052972 |
| Q ≤ 0.1 | 0.281984 | 0.168014 | 0.049502 | 0.349622 | 0.100351 | 0.049502 |
| TA = 25° C, R = 2 cm | | | | | | |
| 0.5 ≤ Q < 1 | 0.817206 | 0.490223 | 0.149801 | 1.008790 | 0.313927 | 0.149801 |
| 0.5 < Q ≤ 0.3 | 0.762105 | 0.472764 | 0.143783 | 0.952108 | 0.301548 | 0.143783 |
| 0.3 < Q ≤ 0.2 | 0.627905 | 0.415840 | 0.125489 | 0.793106 | 0.263418 | 0.125489 |
| Q ≤ 0.1 | 0.551370 | 0.393797 | 0.117604 | 0.703184 | 0.247701 | 0.117604 |

**Tab. 48:** TDE deviation in CM model at different RTML evaluated by D_MAX_ at 25° C

| TDE deviation – CM model - d_MAX_ | | | | | | |
| --- | --- | --- | --- | --- | --- | --- |
| TA = 25° C, R = 0.5 cm | | | | | | |
|  | SP5 | SP3 | SP1 | SP6 | SP4 | SP2 |
| 0.5 ≤ Q < 1 | 0.002750 | 0.001402 | 0.000422 | 0.003270 | 0.000854 | 0.000422 |
| 0.5 < Q ≤ 0.3 | 0.002749 | 0.001457 | 0.000435 | 0.003270 | 0.000880 | 0.000435 |
| 0.3 < Q ≤ 0.2 | 0.002756 | 0.001614 | 0.000477 | 0.003352 | 0.000966 | 0.000477 |
| Q ≤ 0.1 | 0.003079 | 0.002223 | 0.000631 | 0.003883 | 0.001301 | 0.000631 |
| TA = 25° C, R = 1 cm | | | | | | |
| 0.5 ≤ Q < 1 | 0.005468 | 0.002817 | 0.000847 | 0.006589 | 0.001715 | 0.000847 |
| 0.5 < Q ≤ 0.3 | 0.005463 | 0.002932 | 0.000874 | 0.006588 | 0.001769 | 0.000874 |
| 0.3 < Q ≤ 0.2 | 0.005501 | 0.003256 | 0.000960 | 0.006754 | 0.001946 | 0.000960 |
| Q ≤ 0.1 | 0.006167 | 0.004501 | 0.001272 | 0.007819 | 0.002630 | 0.001272 |
| TA = 25° C, R = 2 cm | | | | | | |
| 0.5 ≤ Q < 1 | 0.010808 | 0.006645 | 0.002025 | 0.013374 | 0.004244 | 0.002025 |
| 0.5 < Q ≤ 0.3 | 0.010785 | 0.006928 | 0.002094 | 0.013367 | 0.004395 | 0.002094 |
| 0.3 < Q ≤ 0.2 | 0.010961 | 0.007726 | 0.002312 | 0.013721 | 0.004866 | 0.002312 |
| Q ≤ 0.1 | 0.012373 | 0.010736 | 0.003082 | 0.015851 | 0.006647 | 0.003082 |

**Tab. 49:** TDE deviation in CM model at different RTML evaluated by d_MAX_ at 25° C
